# A Mini‐Spidroin Forms High‐Performance Artificial Spider Silk via Edge‐Cysteine–Locked β‐Sheet Assembly

**DOI:** 10.1002/advs.202517615

**Published:** 2026-01-14

**Authors:** Min Li, Huan Chen, Qi Zhang, Xiaoyun Xu, Yini Zheng, Chunyu Lyu, Weibin Jia, Jun Fan, Fu‐Rong Chen, Wei Liu, Jinlian Hu

**Affiliations:** ^1^ Department of Biomedical Engineering the City University of Hong Kong Hong Kong P. R. China; ^2^ Department of Materials Science and Engineering the City University of Hong Kong Hong Kong P. R. China; ^3^ Shenzhen Key Laboratory For Neuronal Structural Biology Biomedical Research Institute Shenzhen Peking University‐The Hong Kong University of Science and Technology Medical Center Shenzhen P. R. China; ^4^ State Key Laboratory of Transvascular Implantation Devices The Second Affiliated Hospital Zhejiang University School of Medicine Hangzhou P. R. China; ^5^ Institute of Geriatric Medicine Peking University Shenzhen Hospital Shenzhen Peking University‐The Hong Kong University of Science and Technology Medical Center Shenzhen P. R. China

**Keywords:** β‐sheet assembly, disulfide crosslinking, liquid–liquid phase separation, molecular alignment, recombinant spider silk

## Abstract

Spider silk's remarkable mechanical properties arise from its hierarchical organization, where β‐sheet nanocrystals confer strength and amorphous chains impart extensibility. However, replicating this performance in synthetic fibers has been hindered by the challenges of expressing high‐molecular‐weight spidroins and processing them into fibers. Here, we overcome these limitations by engineering a mini‐spidroin (∼33 kDa) that is both easily expressible and spinnable, yet yields fibers with exceptional strength and toughness. Our strategy introduces cysteine residues at the termini of polyalanine (polyA) segments, enabling inter‐strand disulfide bonds that enhance molecular cohesion during liquid–liquid phase separation (LLPS). This “edge‐cysteine locking” promotes directional β‐sheet assembly under extensional flow, resulting in fibers with an ultimate tensile strength of 531 ± 33 MPa and toughness of 182 ± 6 MJ/m^3^, surpassing many bulkier (>100 kDa) recombinant spidroins. Molecular dynamics simulations indicate that disulfide bonds reinforce inter‐strand interactions and prevent chain slippage under shear. By demonstrating that a small, easily produced protein can outperform larger, harder‐to‐process analogs, this work establishes a scalable and efficient route to high‐performance biomimetic fibers, advancing both scientific understanding and practical applications of artificial spider silk.

## Graphical abstract

Cysteine‐engineered mini‐spidroins (∼33 kDa) undergo phosphate‐induced liquid–liquid phase separation (LLPS), where disulfide bonds lock partially aligned polyA segments. Subsequent microfluidic spinning promotes directional β‐sheet crystallization, producing fibers with 531 MPa strength and 182 MJ/m^3^ toughness. This LLPS‐guided design overcomes molecular‐weight limitations, offering a scalable route to robust, high‐performance biomimetic fibers for advanced structural and actuator applications.

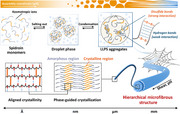

## 1 Introduction

The extraordinary mechanical performance of spider silk (∼1.4 GPa tensile strength, ∼150 MJ m^−3^ toughness) has inspired extensive efforts to engineer synthetic protein‐based materials with comparable properties [1, 2]. These outstanding characteristics originate from a hierarchical microstructure, wherein aligned β‐sheet nanocrystals are dispersed within an amorphous matrix, enabling a unique combination of strength and extensibility [3]. However, replicating this architecture remains a formidable challenge, as natural silk spinning relies on a precisely regulated cascade of molecular events, including spidroin condensation, chain alignment, and controlled crystallization [4, 5, 6]. Recently, liquid–liquid phase separation (LLPS) has been recognized as a critical pre‐assembly mechanism in this process, offering a promising strategy for modulating protein organization and guiding the formation of functional hierarchical materials [7].

LLPS describes the spontaneous demixing of a homogeneous protein solution into two distinct liquid phases, often resulting in the formation of dynamic, protein‐rich condensates [8, 9]. Although early observations of spherical inclusions in spider silk glands date back to the 1980s, in which Vollrath and Knight proposed liquid crystalline [1] and micellar models [10] to explain the supramolecular organization of spidroins, only recently has LLPS been formally recognized in the context of spider silk assembly. Recent studies, particularly by Malay et al., have shown that the modular domain architecture of spidroins responds to specific chemical cues such as pH changes and ionic conditions to initiate LLPS and downstream assembly. The C‐terminal domain (CTD) plays a central role in triggering LLPS, whereas the N‐terminal domain (NTD) undergoes pH‐dependent dimerization that stabilizes intermolecular alignment and promotes fibrillar network formation [7]. The resulting LLPS‐derived condensates are thought to promote local concentration and alignment of aggregation‐prone sequences, thereby facilitating β‐sheet nucleation and hierarchical assembly [11]. Leppert et al. identified tyrosine and arginine residues as key mediators of π–π stacking and electrostatic interactions within these condensates [11], while a recent study further highlights that intrinsically disordered regions in silk proteins also play a crucial role in driving LLPS [12]. Moreover, Eliaz et al. provide NMR evidence showing that LLPS‐mediated intermediates leave lasting structural imprints on the final fiber architecture [13].

While LLPS in spidroin systems is largely governed by multivalent non‐covalent interactions [14], the contribution of covalent modulation remains underexplored. Previous engineering efforts have introduced cysteine residues into recombinant spidroins, either at the CTD or within polyA blocks, to promote disulfide‐mediated crosslinking, primarily aimed at increasing molecular weight and mechanical strength [15, 16, 17]. Recent studies have shown that reversible disulfide bonds formed under redox conditions can modulate molecular assembly in synthetic peptides and proteins [18]. Cysteine‐modified peptides, for example, lower the critical concentration for phase separation [19], and covalent crosslinking has been reported to stabilize condensates and promote ordered protein assembly [20]. These observations raise the possibility that LLPS in spidroin systems may likewise be regulated by redox‐sensitive disulfide bonding. Uncovering this interplay is essential not only for elucidating the molecular basis of silk protein assembly but also for developing strategies to program fiber formation through tunable covalent interactions.

Beyond hierarchical structure, the molecular weight of spidroins plays a critical role in determining the mechanical performance of artificial spider silk. While native dragline silk proteins typically exceed 250 kDa, expressing similarly large constructs in heterologous systems remains difficult due to limitations in current synthetic biology platforms [21, 22]. As a result, most recombinant spidroins fall within the 30–80 kDa range [23, 24, 25]. This raises a key challenge in determining how to impart native‐like fiber mechanics to low‐molecular‐weight constructs. Conventional assembly techniques (such as solvent extrusion, wet spinning, and electrospinning) often rely on high‐concentration processing, which can lead to excessive viscosity and poor spinnability [26]. In contrast, biomimetic strategies that harness LLPS have demonstrated improved nanofibril formation by leveraging shear stress [27], pH gradients [28], or ionic cues [29]. When coupled with LLPS‐mediated pre‐assembly, cysteine engineering at the sequence level enables covalent locking of polyA domains via disulfide bonds. This strategy stabilizes the condensed phase and promotes its transition into highly ordered, mechanically robust fiber architectures. It thus offers a promising route to bridge the performance gap between low‐molecular‐weight recombinant spidroins and their native analogs.

Here, we investigate the introduction of cysteine residues at edge‐specific positions within a ∼33 kDa recombinant spidroin repeat domain. This strategy is intended to promote early‐stage ordered pre‐assembly through disulfide‐mediated interactions under LLPS conditions. We hypothesize that such modification can lower the threshold for phase separation and modulate the subsequent shear‐induced transition of pre‐organized condensates into β‐sheet‐rich fibers. To probe these mechanisms, we combine spectroscopic analysis with molecular dynamics simulations, with a particular focus on β‐strand packing and domain stabilization. Overall, this study aims to evaluate whether rational covalent engineering can endow low‐molecular‐weight spidroins with enhanced assembly behavior and mechanical performance, providing a versatile platform for tunable silk‐based materials.

## 2 Result and Discussion

### 2.1 Cysteine‐Guided LLPS Enables Hierarchical Assembly of High‐Performance Silk Fibers

**FIGURE 1:**
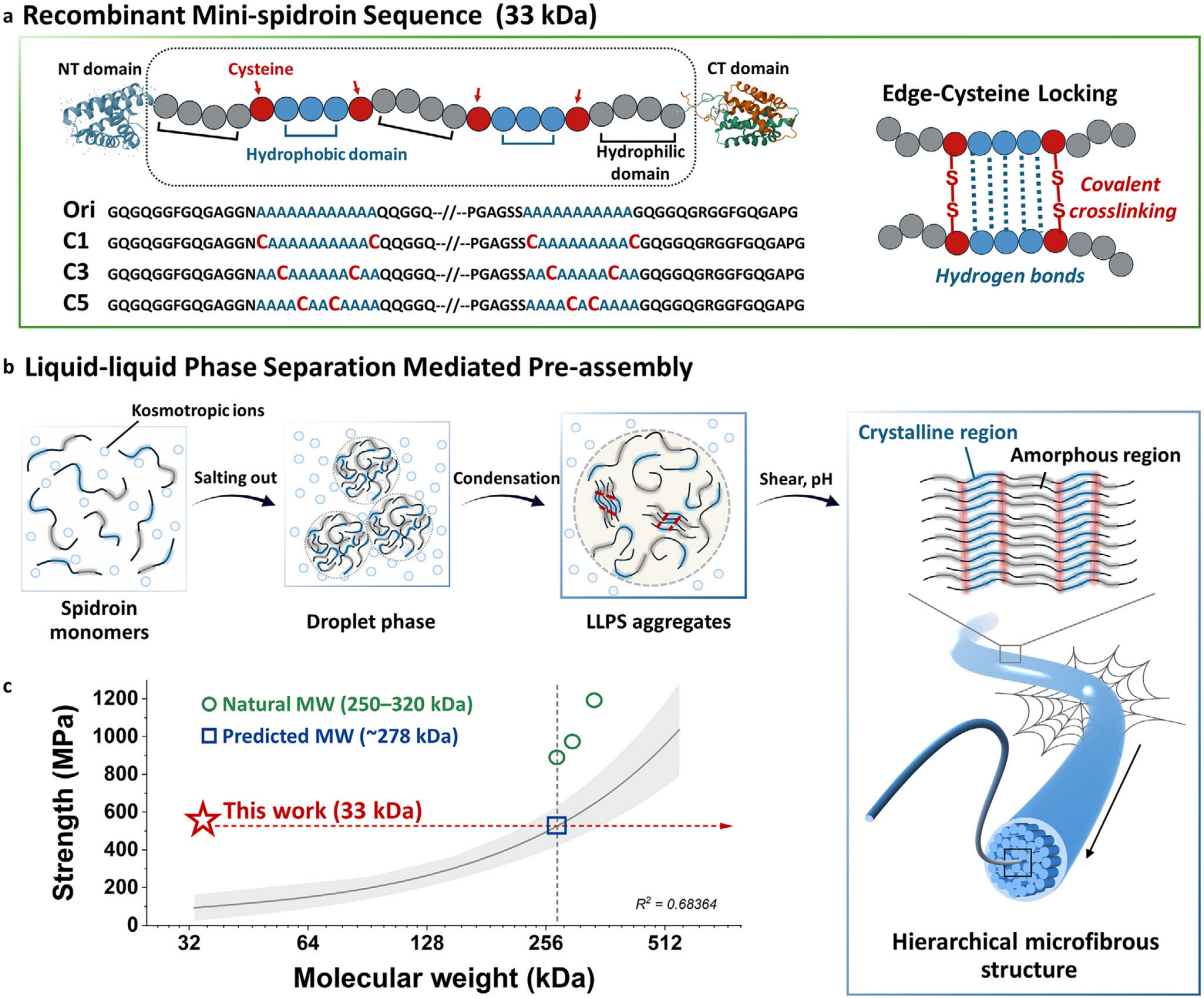
Cysteine‐guided LLPS pre‐assembly enables hierarchical structuring and high‐performance fiber formation. (a) Amino acid sequences of C1, C3, and C5 variants derived from the Ori template, with specific alanine residues substituted by cysteines (highlighted in red). Each construct comprises an NTD, a CTD, and a repetitive region containing two polyA blocks. (b) LLPS‐driven hierarchical assembly: spidroin monomers undergo kosmotropic ion‐induced salting‐out, condense into droplets, and further aggregate via disulfide crosslinking. These dense condensates facilitate β‐sheet nucleation and chain alignment, ultimately crystallizing into microfibrous structures under shear and pH gradients. (c) Strength versus molecular weight of recombinant and natural spider silk fibers (Table S2). The fitted line represents a linear regression of the literature dataset, and the shaded region indicates the 95% confidence interval (R^2^ = 0.6836). Our construct (red) achieves 531 MPa at a molecular weight of 33 kDa, exceeding the value predicted by the literature‐derived molecular weight–strength trend (∼278 kDa, blue) and lying above the range of natural dragline silk (green, 250–320 kDa).

We engineered a series of recombinant mini‐spidroins (∼33 kDa) to explore how sequence‐level modifications regulate hierarchical assembly. The template construct, termed Ori, comprised the repetitive domain of *E. australis* MaSp2 with the NTD from *Euprosthenops australis* MaSp1 and the CTD from *Argiope ventricosus* MiSp [23, 30]. These terminal domains were included to provide the solubility and environmental responsiveness necessary for biomimetic assembly, enabling the preparation of concentrated aqueous dopes and fiber spinning without harsh denaturants or organic solvents. To introduce covalent stabilization near crystalline motifs, we strategically substituted alanine residues at flanking sites of the polyA blocks with cysteines, generating three positional variants (C1, C3, and C5) (Figure 1a; sequences in Table S1). Rosetta calculations [31] predicted lower inter‑chain energies within polyA segments for the cysteine variants (Figure S1), consistent with increased β‑sheet–forming propensity.

Early‐stage assembly was initiated through cysteine‐mediated liquid–liquid phase separation (LLPS), designed to mimic the multistep organization observed in natural spinning. At neutral pH, kosmotropic phosphate ions promoted conformational compaction of repetitive domains, triggering the formation of spherical condensates that gradually matured into dense coacervates (Figure 1b). This suggests that disulfide crosslinks formed between partially aligned polyA segments stabilized these condensates, promoting local β‐sheet–like ordering and establishing a pre‑organized network for subsequent crystallization. These intermediates were then subjected to a low‑pH, high‑shear Y‑shaped microfluidic channel, which drove directional chain alignment and β‑sheet crystallization. Benchmarking against published data shows that the tensile strength of the resulting fibers (531 MPa) surpasses all previously reported mini‐spidroins and lies above the molecular‐weight–strength trend extrapolated from literature (Figure 1c; Table S2). The fitted relationship indicates that achieving a comparable strength would typically require a spidroin of ∼278 kDa, suggesting that LLPS‐mediated pre‐organization can effectively compensate for the intrinsic limitations of low molecular weight. Together, these results demonstrate that motif‐level engineering coupled with LLPS‐assisted assembly provides a practical and scalable route to high‐performance silk fibers derived from low‐molecular‐weight building blocks.

### 2.2 Disulfide‐Mediated LLPS Facilitates Ordered Pre‐Assembly

**FIGURE 2:**
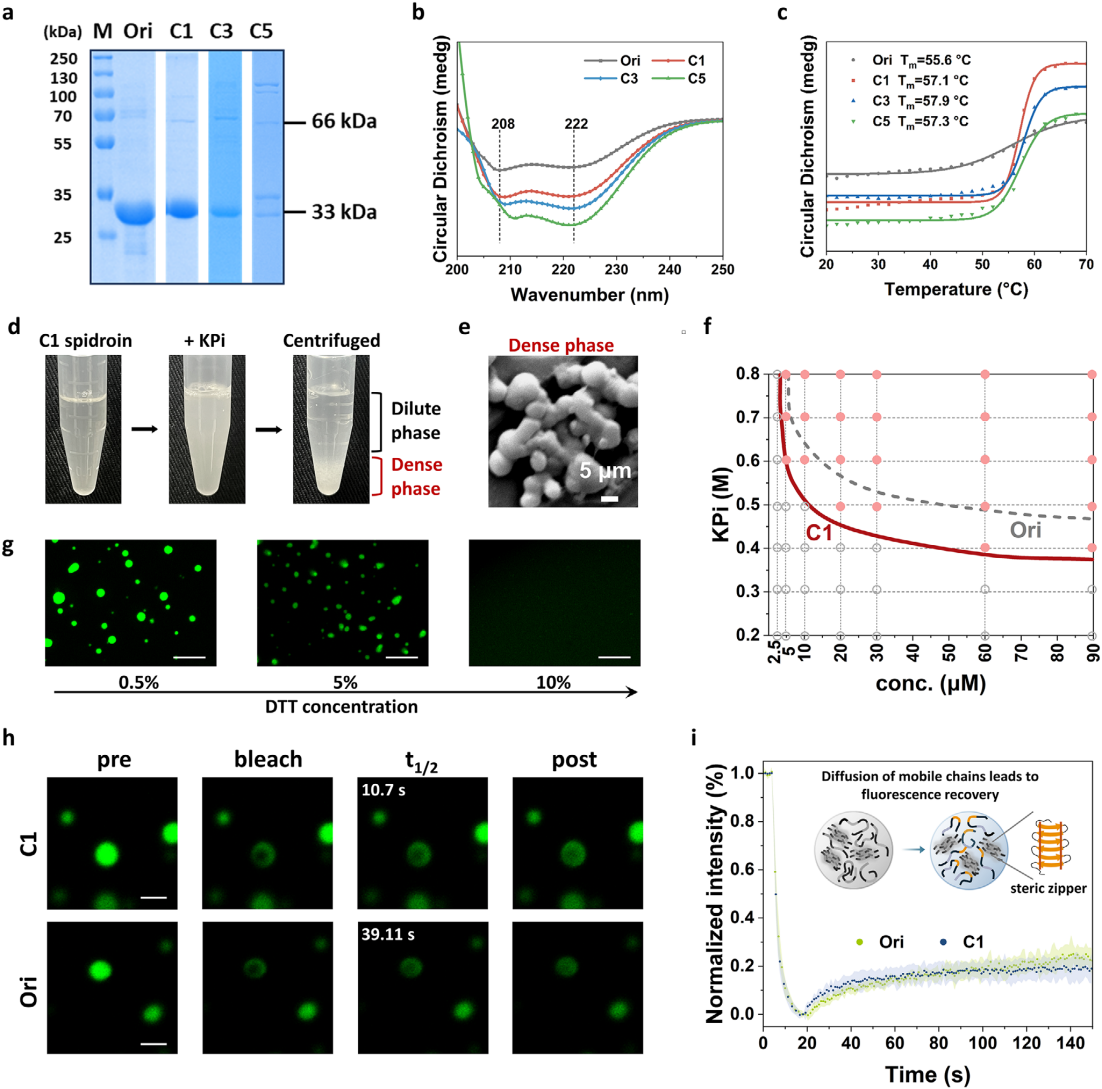
Cysteine‐mediated modulation of LLPS behavior in recombinant spidroins. (a) SDS‐PAGE analysis of spidroin variants (M: molecular weight marker). Ori and C1 exhibit high solubility and show clear bands at ∼33 kDa. (b) CD spectra of Ori, C1, C3, and C5 under reducing conditions, showing prominent α‐helical features with minima at 208 and 222 nm. (c) Thermal unfolding profiles monitored at 222 nm over a temperature range of 25–70°C. (d) LLPS behavior of C1 induced by KPi, forming protein‐rich condensates. (e) SEM image of the high‐density phase of C1 after centrifugation. (f) Phase diagrams constructed by varying protein concentration (x‐axis) and KPi concentration (y‐axis). Microscopic observations were performed at each grid point (dashed lines), and fitted phase boundaries were plotted (red line for C1 variants). (g) Fluorescence images showing inhibition of droplet formation upon DTT treatment, indicating disulfide bond involvement. Scale bar: 20 µm. (h) Representative fluorescence images showing regions of interest for FRAP analysis. Scale bar: 2 µm. (i) FRAP analysis of FITC‐labeled Ori and C1 condensates (150 µm protein, 1.0 m KPi, pH 8.0). Normalized fluorescence recovery curves are plotted over time (*n* = 6), with shaded areas representing standard deviation.

We first evaluated how cysteine placement influences expression and solubility. All mini‐spidroin variants were successfully expressed in *Escherichia coli* and primarily collected in the soluble fraction. Under reducing sodium dodecyl sulfate–polyacrylamide gel electrophoresis (SDS–PAGE) conditions with dithiothreitol (DTT), Ori, C1 and C3 migrated as single bands at approximately 33 kDa, whereas C5 exhibited two closely spaced bands near 33 kDa together with markedly reduced yield, demonstrating that closely spaced cysteine residues disrupt protein stability and promote aggregation (Figures 2a; S2). Size‐exclusion chromatography coupled with multi‐angle light scattering (SEC–MALS) confirmed that Ori, C1, and C3 exist mainly as ∼66 kDa dimers in solution, consistent with the known intermolecular interactions mediated by the CTD (Figure S3). After incubation with oxidized glutathione (GSSG), C1 and C3 displayed additional species near 130 kDa, whereas Ori remained monomeric (Figure S4). DTT treatment collapsed these higher‐molecular‐weight species back to a single 33 kDa band, confirming disulfide‐linked oligomerization. This demonstrated that the introduced cysteines provide additional covalent crosslinking sites that drive oligomer formation under oxidizing conditions. Circular dichroism (CD) spectra of all reduced constructs exhibited the characteristic α‐helical minima at 208 and 222 nm (Figure 2b). C1, C3, and C5 showed a progressive red‐shift of the 208 nm minimum as the spacing between cysteine residues decreased, indicating a systematic increase in local perturbation of the helical environment. In C5, where cysteines were densely clustered, an additional weak shoulder near 205 nm was observed. This feature is consistent with stronger local perturbations of specific helical segments, while the preserved 222 nm minimum confirmed that the global α‐helical scaffold remained intact. Upon oxidation, the cysteine variants exhibited a modest increase in melting temperature (∼1.5°C above Ori; Figures 2c; S5), consistent with slightly reduced backbone flexibility due to disulfide bond formation.

We next investigated how disulfide bonding influenced LLPS behavior under gland‐mimicking conditions. Upon addition of potassium phosphate (KPi, pH > 7.0), C1 solutions became turbid and rapidly underwent phase separation (Figure 2d). Centrifugation yielded a nearly transparent supernatant and a dense protein‐rich coacervate phase (Figure 2e). Phase diagrams showed that C1 had a substantially lower LLPS threshold compared to Ori. For instance, at 0.5 M KPi, C1 formed droplets at 10 µm (∼0.33 mg/mL), whereas Ori required ∼60 µm (Figure 2f). Increasing concentrations of DTT resulted in progressive disintegration of C1 droplets (Figure 2g), confirming that redox‐sensitive disulfide bonds stabilized C1 condensates. C1 condensates displayed distinct morphological and dynamic features. Droplet growth was accelerated, with diameters nearly twice those of Ori after 30 min (Figure S6), and C1 solutions showed an earlier onset of turbidity and a thermally induced sol–gel transition (Figure S7a,b). Fluorescence recovery after photobleaching (FRAP) revealed a lower mobile fraction (∼20%) relative to Ori (Figure 2h) and a significantly shorter half‐recovery time (t_1/2_ = 10.7 s vs. 39.1 s; Figure 2i), indicative of enhanced intermolecular interactions yet rapid network reconfiguration [7, 29, 32]. Although the presence of rigid β‐sheets could not be directly confirmed at this stage due to the turbidity and structural heterogeneity of the condensates, the combination of compact architecture and dynamic rearrangement suggests that disulfide‐mediated LLPS promoted early ordering within the polyA‐rich domains, thereby priming the system for directional β‐sheet crystallization upon subsequent shear and acidification.

### 2.3 Acidification Triggers Nanofibril Formation from Condensates

**FIGURE 3:**
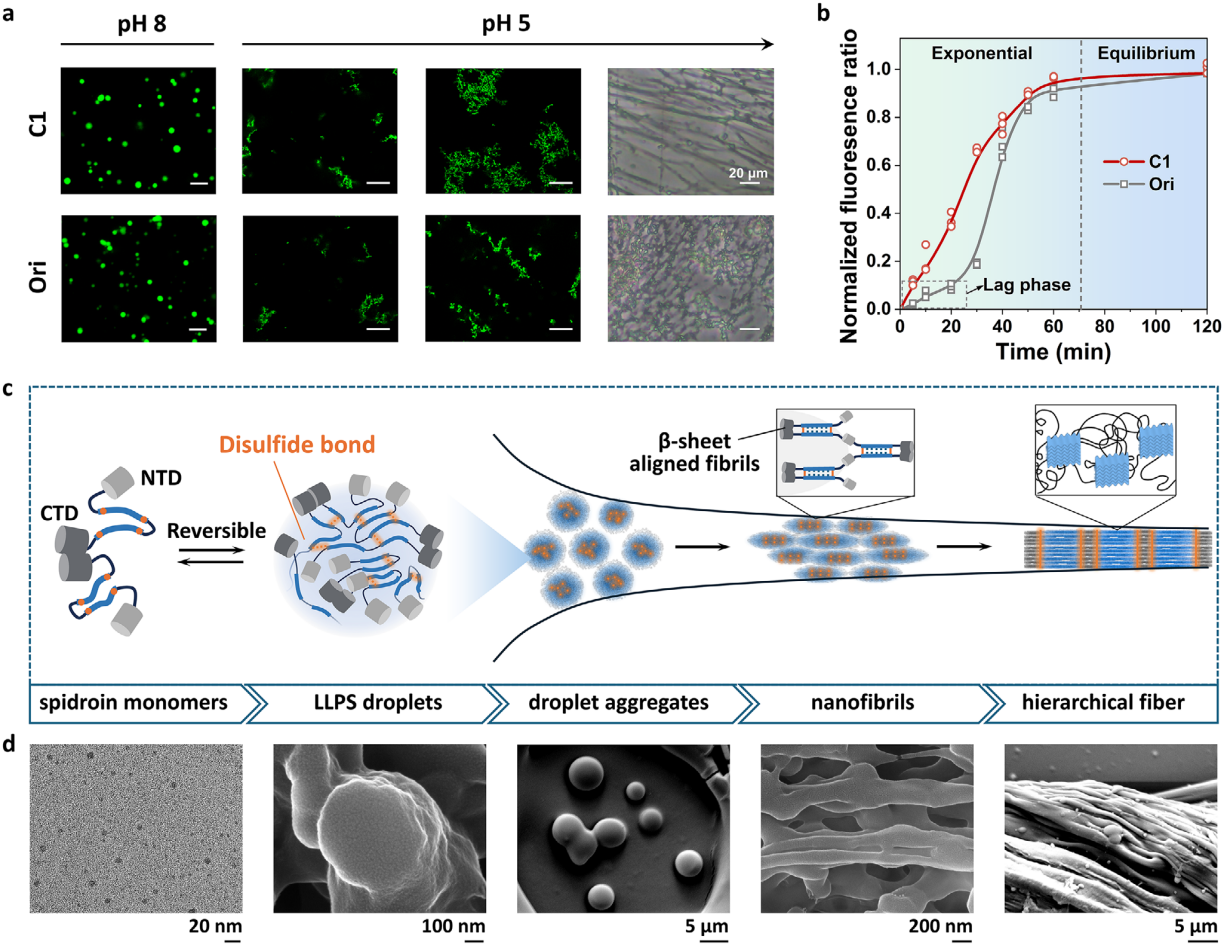
Droplet‐to‐nanofibril transition of cysteine‐engineered spidroins via LLPS and shear in biomimetic spinning. (a) C1 forms stable LLPS droplets at pH 8. Upon acidification to pH 5, the droplets undergo spherical aggregation and gradually evolve into interconnected fibrous networks (Optical images). Scale bar = 5 µm. (b) Time‐resolved ThT fluorescence showing a shorter lag phase in C1 condensates. (c) Schematic representation of LLPS‐guided pre‐assembly mediated by reversible disulfide interactions and subsequent shear‐ and pH‐induced β‐sheet alignment during biomimetic spinning. (d) TEM and SEM images illustrating the transition from monomers to aligned, hierarchically structured microfibers.

Acidification drove a strikingly different transition of condensates into fibrillar structures. At pH 5.0, C1 droplets progressively reorganized into interconnected submicron networks with distinct fibrillar morphologies, whereas Ori formed amorphous, irregular aggregates lacking structural continuity (Figure 3a). This distinction became increasingly evident over time as C1 assembled into coherent fibrillar architectures, suggesting a more efficient structural reorganization. Thioflavin T (ThT) fluorescence revealed distinct early‐stage kinetics between the variants. C1 displayed an immediate fluorescence increase, whereas Ori exhibited a pronounced lag phase before signal development (Figure 3b), and the endpoint ThT measurements confirmed higher β‐sheet content in C1 condensates (Figure S7c).

This acid‑triggered transformation generated a cohesive and viscoelastic phase suitable for direct fiber drawing. When 0.5 µL of concentrated spidroin solution was exposed to a 1.0 M KPi buffer (pH 5.0), dense condensates rapidly formed at the interface, from which continuous fibers (∼5 cm in length, ∼50 µm diameter) could be drawn manually using a fine syringe needle (Figure S8). Using the Y‐shaped microfluidic platform, we further resolved the droplet‐to‐nanofibril transition by systematically tuning the flow‐rate ratio between the spidroin stream and phosphate buffer (Figures 3c, d). In this configuration, the two streams converged within the PDMS junction, where exposure to kosmotropic phosphate ions triggered LLPS and generated dense condensates. These condensates were subsequently elongated under extensional flow, revealing a continuous structural evolution from dispersed droplets to aligned nanofibrillar assemblies. In dilute aqueous solution, C1 existed as 3–8 nm species, which assembled into LLPS droplets as protein concentration increased and phosphate diffused into the stream. At 5% (w/v) protein concentration, the KPi/spidroin flow‐rate ratio precisely controlled condensate morphology. At a ratio of 0.25, dispersed nanodroplets (100–500 nm) formed and subsequently fused into larger coacervates (5–20 µm). As the ratio increased, droplet proximity and partial fusion intensified, and under simultaneous extensional flow, some droplets transiently exhibited bead‐on‐a‐string‐like intermediates (Figure S9). These features likely reflected incomplete coalescence during axial deformation, producing bead‐like domains linked by thin bridges. When the flow‐rate ratio reached 1, the condensed droplets reorganized into aligned nanofibrils under the combined influence of acidification and shear, and these nanofibrils could be directly collected in the coagulation bath. Increasing the protein concentration to 15% (w/v) yielded continuous hierarchical fiber assemblies, demonstrating that both dope concentration and flow‐induced confinement modulated the structural evolution of LLPS condensates in the microfluidic environment. This hierarchical assembly pathway (Figure 3d) integrates sequential organizational cues: disulfide‑mediated LLPS enables spatial confinement, acidification triggers β‑sheet ordering, and mechanical drawing locks in axial alignment. The resulting uniaxially aligned architecture closely resembled native spider silk [33, 34, 35], highlighting how cysteine‑driven condensate reorganization effectively primes the system for directional nanofibril assembly and macroscopic fiber formation.

### 2.4 Enhanced Mechanical Performance via Hierarchical Stabilization

**FIGURE 4:**
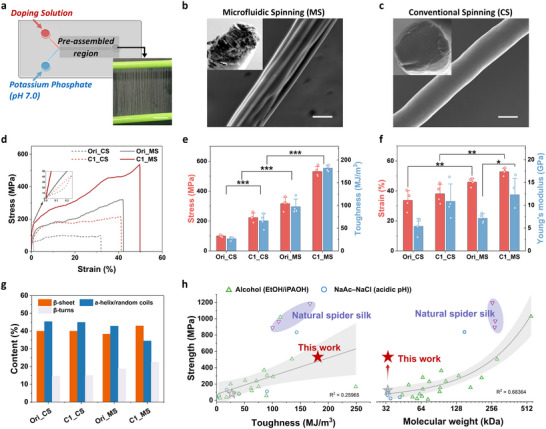
Enhanced mechanical performance of C1 fibers via LLPS‐guided microfluidic spinning. (a) Microfluidic spinning (MS) process integrating LLPS‐driven molecular pre‐assembly. The inset shows bulk fiber collection using a rotating mandrel. (b) SEM images of C1_MS fibers reveal denser morphology with distinct hierarchical substructures. (c) SEM images of C1_CS fibers display smooth surfaces and uniform cross‐sections. Scale bars: 5 µm (top), 20 µm (bottom). (d) Representative stress–strain curves of four fiber types derived from distinct protein designs and processing methods, with C1_MS fibers exhibiting superior strength and extensibility (n = 4, Table S3). (e) Comparison of tensile strength and toughness. (f) Comparison of elongation at break and Young's modulus. (g) FTIR‐derived secondary structure compositions, showing elevated β‐sheet content in C1‐based fibers. (h) Benchmark plots comparing the relationships between tensile strength and toughness (left; R^2^ = 0.26) and tensile strength and molecular weight (right; R^2^ = 0.68) for natural and recombinant spider silk fibers. Shaded regions indicate the 95% confidence intervals of linear regression fits. Recombinant fibers are grouped based on their processing conditions, with one group produced by alcohol‐based coagulation (e.g., ethanol or isopropanol), which typically disrupts native‐like structural features, and the other produced under aqueous acidic spinning conditions (e.g., NaAc–NaCl).

Fibers were prepared using two spinning routes in order to compare LLPS‐guided assembly with a conventional process. To translate the droplet‐to‐nanofibril transitions described above into a continuous spinning process, the engineered C1 spidroin (33 kDa), comprising the NTD, CTD, and site‐specific cysteines, was formulated into stable aqueous dopes at concentrations up to 20%. In the microfluidic spinning route, the dope entered a channel where controlled mixing with 0.3 m phosphate buffer (pH 7.0) initiated the formation of LLPS droplets. As the mixture progressed through the pre‐assembly region and reached the lower‐pH outlet, it experienced an acidification step analogous to that of the natural spinning duct, which promoted the consolidation of the condensates into fibrillar networks. This process enabled the continuous production of ∼150 m of fibers from only 200 mg of protein (Figure 4a). A conventional wet‐spinning route was also employed, in which concentrated protein dopes were extruded into the same coagulation bath and subsequently drawn into fibers. Post‐stretching (1–3×) in an oxidizing medium further improved molecular alignment, as evidenced by birefringence under polarized light microscopy (Figure S10). Hierarchical structural organization was a distinguishing feature of C1 fibers produced by microfluidic spinning (C1_MS). SEM revealed uniform diameters (∼16 µm), highly aligned nanofibrils (∼200 nm), and bundled core substructures (Figure 4b). In contrast, C1 fibers obtained through conventional spinning (C1_CS) displayed smooth surfaces and compact interiors lacking fibrillar organization (Figure 4c), underscoring the importance of LLPS‑guided pre‑assembly in generating multiscale architecture [27, 29].

Mechanical testing showed that C1_MS fibers reached a tensile strength of 531 ± 33 MPa, toughness of 182 ± 6 MJ m^−3^, and Young's modulus of 12.3 ± 3.6 GPa, representing 2.4‑fold, 2.8‑fold, and 1.1‑fold increases over C1_CS fibers (224 ± 29 MPa, 66 ± 15 MJ m^−3^, and 10.9 ± 3.8 GPa). Ori fibers also benefited from microfluidic spinning, with Ori_MS outperforming Ori_CS, supporting the contribution of LLPS‐guided hierarchical organization to mechanical performance (Figures 4d; S11). Moreover, C1_MS exhibited approximately 1.7‐fold higher strength and 1.9‐fold higher toughness than Ori_MS, demonstrating that sequence engineering and LLPS act synergistically to reinforce hierarchical structure (Figure 4e). Despite being derived from constructs nearly tenfold smaller in molecular weight than native spidroins, C1_MS fibers achieved a toughness of 182 MJ/m^3^ (Figure 4f), exceeding that of *Nephila clavipes* dragline silk. The influence of disulfide linkages on fiber mechanics was examined by testing fibers treated with DTT or prepared without H_2_O_2_ (Figure S12). Under reducing conditions, the solvent accessible disulfide bonds were expected to be cleaved, while more buried cross‐links may remain intact. Both reduced C1_CS and C1_MS fibers showed roughly twofold decreases in strength relative to oxidized samples, along with significant reductions in modulus and toughness. These changes indicate that disulfide linkages formed under oxidizing conditions played an important role in maintaining mechanical stability and delaying chain slippage. For C1_MS fibers, removing H_2_O_2_ from the coagulation bath caused a more moderate decrease in strength (∼400 MPa) and toughness (∼88 MJ/m^3^), likely due to limited disulfide formation supported by ambient oxygen during drying. These results showed that disulfide bonding facilitated by oxidative conditions contributes to the mechanical reinforcement of both constructs.

The amide I band (1700–1600 cm^−1^), arising from C═O stretching vibrations of the peptide backbone, was analyzed by FTIR spectroscopy to evaluate the secondary structures of silk proteins. According to previous reports [25, 36], peaks at 1618–1633 cm^−1^ were assigned to β‐sheets, while those at 1640 cm^−1^, 1655 cm^−1^, and 1661 cm^−1^ corresponded to random coils and α‐helices, respectively. Additional peaks at 1670 cm^−1^, 1682 cm^−1^, and 1696 cm^−1^ were attributed to β‐turns. The spectrum of C1_MS fibers displayed a pronounced β‐sheet peak at 1620 cm^−1^ (Figure S13a). Quantitative analysis further revealed increased β‐sheet (∼44%) and β‐turn (∼23%) contents compared with C1_CS and Ori_MS fibers (Figures 4g; S13b), indicating a substantial conformational shift from α‐helix/random‐coil to β‐sheet structures. This hierarchical ordering was closely associated with the exceptional mechanical resilience of C1_MS fibers, whose toughness exceeded that of native dragline silk (Figure 4h). Previous strategies to improve recombinant silk mechanics had primarily focused on increasing molecular weight through extended repetitive sequences, which imposed significant challenges for heterologous expression [17, 37]. Moreover, conventional spinning methods often require organic solvents or alcohol‑rich coagulation baths that disrupt native‑like conformations. In contrast, our fully aqueous strategy relied on a 33 kDa construct whose site‑specific cysteines drove LLPS‑mediated condensation, directional alignment, and disulfide‑guided stabilization. This biomimetic assembly pathway enabled early‑stage pre‑organization and hierarchical locking, offering a scalable and environmentally benign approach for producing mechanically robust fibers with native‑like architectures.

### 2.5 Disulfide Locking Enhances β‐Sheet Alignment and Cohesion

**FIGURE 5:**
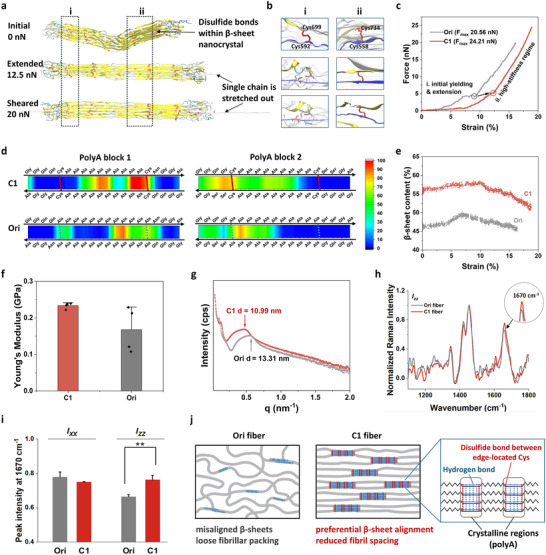
Molecular and structural features of disulfide‐stabilized β‐sheet crystalline in C1 fibers. (a) Representative conformations of C1 spidroin under increasing shear, with secondary structures shown in New Cartoon style: β‐sheets (yellow), β‐turns (cyan), random coils (white), and α‐helices (blue); disulfide‐forming cysteines are rendered in tube style. (b) Enlarged views of regions i and ii from (a), highlighting disulfide bridges between adjacent β‐strands. PolyA‐flanking disulfide bonds rupture earlier under tensile load. (c) Representative force–strain curves for C1 and Ori models under simulated tension. (d) Heatmaps of β‐sheet probability at the residue level in the two polyA blocks during the final 15 ns of simulation. (e) Evolution of β‐sheet content during tensile deformation. (f) AFM showing spatial distribution of Young's modulus across C1 and Ori fiber surfaces. (g) SAXS profiles indicate more compact packing in C1 fibers. (h) Polarized Raman spectra and (i) intensity ratios at 1670 cm^−1^ (*I_zz_* orientation) demonstrating improved β‐sheet alignment in C1. (j) Schematic representation of the experimentally supported mechanism by which edge‐localized disulfides enhance β‐sheet stability and alignment in C1 fibers.

All‐atom molecular dynamics (MD) simulations provided molecular‐level insights into how disulfide crosslinking may influence the mechanical stability of β‐sheet nanocrystals. Cysteine residues in the C1 model were placed at both termini of the polyA segment, which enabled the modeling of disulfide bonds at terminal (region i) and central (region ii) positions within a 3 × 5 antiparallel polyA lattice (Figures 5a; S14). Incremental chain pulling suggested that terminal disulfides tended to rupture earlier, whereas central disulfides sustained forces approaching 20 nN (Figure 5b). The force‐displacement response of C1 showed a higher yield threshold and a rupture force of 23.7 nN, 13% greater than the cysteine‑free Ori control (Figures 5c; S15). This trend was consistent with a potential role of interior disulfides in reducing early chain sliding and preserving β‑sheet alignment under load. The β‑propensity heatmaps supported a preferential retention of β‑sheet structure around the central crosslinking sites (Figure 5d). A brief equilibrium comparison of the two systems without pulling (Figure S16) showed a similar trend in the baseline stability of the β sheet domains. The overall β‑content in C1 increased from 56% to 63% during the initial 10% deformation (Figure 5e), suggesting that disulfide anchoring favors strain‑induced ordering rather than disruption.

This disulfide‐mediated stabilization was further reflected in the hierarchical organization of the fiber matrix. Atomic force microscopy (AFM) phase imaging revealed a homogeneous morphology in C1 with low phase contrast, consistent with a well‑integrated crystalline–amorphous network (Figure S17a). Ori fibers, in contrast, displayed fragmented crystalline domains dispersed in a less ordered matrix. Surface roughness of C1 (Rq = 94 nm, Ra = 79 nm) was nearly 50% lower than Ori, reflecting a smoother surface topology (Figure S17b). AFM nano‑indentation confirmed greater mechanical homogeneity in C1, showing higher average modulus and reduced spatial variation (Figure 5f), indicative of continuous stress propagation through a cohesive network. At the nanoscale, disulfide crosslinking induced a more compact and aligned internal structure. Small‑angle X‑ray scattering (SAXS) revealed a shift in the lamellar scattering peak from 0.47 nm^−1^ in Ori to 0.57 nm^−1^ in C1, corresponding to a 17% decrease in inter‑lamellar spacing (Figure 5g). Polarized Raman spectra showed a more intense β‑sheet band at 1670 cm^−1^ under the *I_zz_* orientation in C1 fibers, reflecting enhanced alignment along the fiber axis (Figures 5h,i; S18). Together, these findings indicated that site‐specific disulfide crosslinking contributes to the stabilization of β sheet nanocrystals during LLPS‐mediated pre‐assembly and supported their axial alignment under extensional flow. This behavior was consistent with the increase in lamellar compactness, the improved continuity at the crystalline–amorphous boundary, and the more uniform transfer of stress across structural levels (Figure 5j). These features resemble organizational motifs found in native spider silk and suggest that the same design principle may help improve the structural integrity and mechanical consistency of engineered protein‐based fibers.

## 3 Conclusions

This study presents an approach that integrates motif‑targeted protein engineering with LLPS‑mediated pre‑assembly to obtain mechanically robust recombinant silk fibers. Introducing cysteine residues into the polyA domain enabled low‑molecular‑weight (∼33 kDa) spidroins to form fibers with tensile strength and toughness up to 531 MPa and 182 MJ/m^3^, values supported by mechanical testing. Structural characterizations, including AFM, SAXS, and polarized Raman spectroscopy, indicated that C1 fibers possessed denser lamellar packing, smoother surface morphology, and more homogeneous stress distribution than their cysteine‑free counterparts. Failure‐related analyses, together with supporting trends from molecular dynamics simulations, suggested that disulfide crosslinks help reduce chain slippage and enhance cohesion at the crystalline–amorphous interface, consistent with the observed improvements in mechanical performance.

Compared with approaches that rely on high molecular weight constructs or organic solvent‐based coagulation systems, this aqueous and biomimetic pathway reflects several features of native silk spinning, including LLPS‐driven confinement, acid‐induced β‐sheet ordering, and flow‐mediated alignment. These results supported a modular strategy for constructing hierarchical protein‐based fibers from genetically tractable, low molecular weight building blocks. The same design principles may be extended to the development of proteinaceous materials requiring controlled structural organization and reliable mechanical properties.

## Author Contributions

Prof. J.H. conceived the idea, initiated the project, designed experiments related to fiber fabrication, and provided guidance throughout the manuscript development. M.L. conducted the majority of the experiments and drafted the manuscript. H.C. carried out the molecular dynamics simulations and contributed to manuscript writing. Q.Z. participated in protein purification. X.X. contributed to the wet spinning process. Y.Z. conducted the TEM characterization. C.L. assisted in the design of spidroin variants. Dr. W.J. assisted with mechanical testing. Dr. J.F. supervised the simulation experiments. Prof. W.L. provided guidance on genetic engineering. Prof. F.‐R.C. supervised TEM imaging and analysis.

## Conflicts of Interest

The authors declare no conflict of interest.

## Supporting information




**Supporting File**: advs73456‐sup‐0001‐SuppMat.docx.

## Data Availability

The data that support the findings of this study are available in the supplementary material of this article.
