# Supplementary material for: A Mini‐Spidroin Forms High‐Performance Artificial Spider Silk via Edge‐Cysteine–Locked β‐Sheet Assembly
Source: Adv Sci (Weinh). 2026 Jan 14;13(10):e17615. doi: 10.1002/advs.202517615 (PMC12915095; doi:10.1002/advs.202517615)
Supplement: Supplementary file 1 — Supporting File: advs73456‐sup‐0001‐SuppMat.docx. [file ADVS-13-e17615-s001.docx]

Supporting Information

A Mini-Spidroin Forms High-Performance Artificial Spider Silk via Edge-Cysteine–Locked β-Sheet Assembly

Min Li, Huan Chen, Qi Zhang, Xiaoyun Xu, Yini Zheng, Chunyu Lv, Weibin Jia, Jun Fan, Fu-Rong Chen, Wei Liu^*^, Jinlian Hu^*^

**Experimental Sections**

*Designed Spidroin Variant:* A synthetic gene encoding NT domain of MaSp1 sequence of E. australis (EMBL No. AM259067), a repetitive module containing two polyA and glycine-rich motifs of E. australis MaSp2 (GenBank: CAM32272.1), and a CT domain derived from MiSp sequence of A. ventricosus (GenBank No. JX513956) was synthesized ^[1]^ and delivered on plasmid pET32M_NT2RpCT_Ori term from GenScript Corporation (Nanjing, China). The engineered variant was designed to contain amino acid residue substitutions, specifically, two pairs of cysteines (C) were replaced with alanines (A) at specific sites of two polyA blocks (positions 148&159, 183&193; 150&157, 185&191; 152&155, 187&189) using two-step site-directed mutagenesis and homologous recombination approach. For cloning experiments, the Phanta Max Super-Fidelity DNA Polymerase (Vazyme Biotech Co., Ltd.) and TIANprep Mini Plasmid Kit (TIANGEN Biotech Co., Ltd.) were utilized. The purities and sizes of the variant were assessed using 1% agarose gel for electrophoresis. DNA sequencing was verified at Biotech Bioengineering (Shanghai) Co. The constructs with two pairs of Ala to Cys mutations were named C1, C3, and C5, respectively, while the NT and CT regions remained unchanged.

*Expression and Purification of Recombinant Spidroin:* The purification procedure for recombinant spidroin C1, C3 and C5 was conducted as previously reported.^[1]^ Briefly, the constructed plasmid was transformed into *E. coli*. BL21 and amplified in culture. Following induction with 0.25 mM isopropyl β-D-1-thiogalactopyranoside (final concentration), the cells were harvested by centrifugation at 5,000 rpm. After lysis in a high-pressure cell disrupter at 600-1000 Psi, the supernatant was firstly purified by Ni-immobilized metal affinity (IMAC) column (GE Healthcare, USA), and the protein was eluted in 20 mm Tris-HCl containing 500 mM imidazole. The eluates were then digested with excess HRV 3C protease at 4 °C overnight, followed by re-purification using a HiLoad 26/600 Superdex 200 pg column (GE Healthcare). Finally, the proteins were concentrated in ultrafiltration tubes (10 kDa MWCO, Millipore™, Germany) and stored at −20°C for further use.

*SDS–PAGE Analysis:* Protein samples were analyzed by SDS–PAGE using 12% Tris–Glycine precast gels. For reducing conditions, samples containing 100 mM DTT were mixed with 5× loading buffer and heated at 95 °C for 5 min to ensure complete denaturation and reduction of disulfide bonds. For oxidizing conditions, loading buffer was prepared without reducing agents (DTT or β-mercaptoethanol). Protein samples were first incubated overnight at 4 °C in a solution containing excess GSSG to promote disulfide formation, followed by addition of the non-reducing loading buffer and heating at 95 °C for 5 min. For reduced-after-oxidation controls, oxidized samples were subsequently treated with DTT prior to heating.

*SEC–MALS Analysis:* Protein samples prepared in 50 mM Tris–HCl (pH 7.5), 100 mM NaCl, 1 mM EDTA, and 1 mM DTT were analyzed using an ÄKTA FPLC system equipped with a Superdex 200 Increase 10/300 GL column (GE Healthcare). Elution was monitored with a multi-angle light scattering detector, and molecular weights were determined using ASTRA 6 software (Wyatt Technology).

*Circular Dichroism:* Protein concentration of 50 μM were measured in a 200 μL quartz cuvette using a Chirascan CD spectrometer (Applied Photophysics, UK). Far-UV spectra were recorded from 280 to 190 nm. Samples for reducing measurements contained 100 mM DTT. Samples for oxidizing measurements were pre-incubated overnight at 4 °C with excess GSSG. Temperature scans were conducted in the range of 24-70°C at a 2 °C ramping gradient, and thermal unfolding curves were obtained by monitoring the molar residual ellipticity ([θ], deg cm^2^ dmol^−1^) at 222 nm.

*Spidroin Self-assembly:* Ori, C1, and C3 samples were prepared in 20 mM Tris buffer at pH 8. Assembly was initiated by adding potassium phosphate at pH 8 to a final concentration of 50 mM at 20 °C. Turbidity at 340 nm was monitored every 10 minutes in triplicate using a UV/Vis spectrophotometer. For thermal ramping experiments, 200 μL aliquots were heated from 18 °C to 60 °C at a rate of 2 °C per minute, and absorbance at 600 nm was recorded at one-minute intervals.

*Phase Separation Maps*: LLPS behaviour was evaluated using protein concentrations from 2.5 μM to 90 μM and potassium phosphate concentrations from 0.2 M to 0.8 M. Mixtures of 20 μL were prepared in PCR tubes at 25 °C. A 5‑μL aliquot was transferred to a glass slide and observed immediately by optical microscopy to determine whether LLPS droplets formed. Each condition was recorded as positive or negative for LLPS and plotted on protein‑versus‑phosphate grids. Boundary curves were drawn manually. All conditions were tested at least twice, and replicates produced consistent results.

*Fluorescent Labeling of Spidroins:* Lyophilized spidroins were dissolved in deionized water and subjected to buffer exchange using Amicon Ultra centrifugal filters (10 kDa MWCO) to remove residual Tris and concentrate the protein to 5 mg/mL. The protein was then reacted with fluorescein isothiocyanate (FITC) at a mass ratio of 50:1 (protein: FITC) for 8 h at 4 °C in the dark. To quench unreacted FITC, ammonium chloride was added to a final concentration of 50 mM, followed by a 2 h incubation at 4 °C. Labeled protein was purified using a Sephadex G-25 desalting column and centrifuged at 8000 × g for 2 min.

*Confocal Laser Scanning Microscopy:* Fluorescence images were acquired on a Zeiss LSM 880 confocal microscope using a 448 nm laser and 40×/1.3 NA or 63×/1.4 NA oil immersion objectives. For DTT-induced phase separation, 20 mM C1 samples were mixed with equal volumes of DTT to obtain final concentrations of 0.5%, 5%, or 10%, incubated for 30 seconds, and transferred to a glass-bottom dish for imaging. For pH-induced phase separation, 20 mM protein samples were adjusted to approximately pH 5.0 by mixing with 1 M potassium phosphate at pH 5.0 in a 1:4 ratio. Samples prepared under the same conditions using 1 M potassium phosphate at pH 8.0 served as the control. Fluorescence images were recorded immediately within the first 30 seconds and again after 10 minutes, and optical microscopy images were acquired after 30 minutes under the same conditions.

*Fluorescence Recovery After Photobleaching (FRAP):* FRAP measurements were performed on FITC-labeled spidroin (5 mg/mL, unlabeled: labeled = 2:1) mixed with 1.0 M KPi (pH 8.0) in a custom glass-bottom dish. Spherical droplets (~10× bleach spot diameter) resting on the dish surface were selected. A 0.55 μm region was photobleached using a 63×/1.4 NA objective, and fluorescence recovery was monitored every ~3 s for 150 s. Representative images were acquired both before photobleaching and at selected recovery time points. Three regions of interest (bleached area, whole droplet, and background) were analyzed using Zen (Zeiss). Results from six independent experiments (N = 6) were fitted with a biexponential model using easyFRAP ^[2]^ to extract mobile fractions and half-time of recovery (t_1/2_).

*ThT fluorescence Spectrometry Analysis:* The protein sample was transferred to a 96-well plate with black walls and transparent bottom to a final volume of 200 μL. Thioflavin T (ThT) was added to a final concentration of 20 × 10^-6^ M and incubated at room temperature for 36 h. After a 10-second equilibration period, fluorescence was monitored at an excitation wavelength of 435 nm and recorded the emission spectra between 400 and 600 nm.

*Biomimetic Fiber Formation:* Condensed protein samples were prepared by pipetting 0.5 μL of concentrated spidroin solution onto a glass slide, followed by gently overlaying a 5 μL drop of 1.0 M KPi buffer (pH 5.0). The mixture was allowed to equilibrate for several seconds until a condensed protein phase formed. Continuous fibers were then manually drawn using a fine syringe needle under ambient conditions.

*Microfluidic Device*: A Y-shaped microfluidic chip was fabricated using standard soft-lithography. The channel mold was prepared by photolithographic patterning of SU-8 photoresist (SU-8 3050) on a silicon wafer, followed by overnight silanization with trichloro(1H,1H,2H,2H-perfluorooctyl)silane. Polydimethylsiloxane (PDMS; SYLGARD 184) was cast onto the mold, cured, and peeled off to generate the channel-bearing slab. Two inlet ports and one outlet port (∼300 μm diameter) were punched. The inlet channels (2 cm each) intersected at a 60° angle, and the outlet channel was 4 cm long. The PDMS slab was placed onto a glass coverslip without permanent bonding and inspected for leakage before use.

*Microfluidic Spinning (MS):* Spidroin dopes (5–20% w/v) and potassium phosphate buffer (30 mM, pH 8.0) were introduced into the two inlets of the microfluidic device at flow rates of 0.02–0.1 mL h^-1^ and 0.1–0.4 mL h^-1^, respectively. The outlet channel was connected to a 30 G needle, enabling continuous extrusion of the forming filament directly into a coagulation bath composed of 500 mM sodium acetate and 200 mM NaCl (pH 5.0). To observe the droplet-to-nanofibril transition, a 5% (w/v) spidroin solution was used while varying the flow-rate ratio of KPi/spidroin from 0.25 to 1. After spinning, the PDMS slab was gently removed, leaving droplets or nascent filaments adhered to the coverslip. Following complete drying, coverslips were mounted on aluminum stubs with conductive carbon tape, sputter-coated with gold for 2 min, and imaged using a JEOL-6000 scanning electron microscope operating at 15 kV.

Fibers used for mechanical testing were produced from 20% (w/v) spidroin dopes at a KPi/spidroin flow-rate ratio of 1. The as-spun fibers were stretched to 200% of their original length in the same coagulation bath supplemented with 20% H_2_O_2_ and allowed to solidify for 10 min before collection. A coagulation bath without H_2_O_2_ was used to prepare the corresponding control fibers.

*Conventional Spinning (CS):* Purified spidroin solutions (20–25% w/v, C1 or Ori) were loaded into a 1 mL syringe fitted with a 30 G steel needle (inner diameter 0.15 mm). The dope was extruded at a constant flow rate of 17 μL min^-1^ into a coagulation bath composed of 500 mM sodium acetate and 200 mM NaCl (pH 5.0). The as-spun fibers were stretched to 300% of their initial length in the same bath supplemented with 20% H_2_O_2_, allowed to solidify for 10 min, and then air-dried at room temperature. Ori fibers were prepared in parallel under identical conditions and used as controls.

*Mechanical Analysis of Fibers:* Oxidized fibers and oxidized fibers subjected to reduction in a DTT containing coagulation bath were prepared for mechanical testing. General tensile tests were performed on a 5942-Instron tensile testing machine with a 50 N load cell. Fiber diameters were assessed at five distinct locations along each fiber using an optical microscope, and the average diameter was subsequently calculated. The fibers were fixed at a length of 20 mm using double-sided tape and subjected to tension at a rate of 5 mm/min until failure.

*Atomic Force Microscopy:* Surface morphology characterization was performed using an atomic force microscope (Dimension Icon, Bruker, Germany) in tapping mode. Individual fibers obtained from microfluidic spinning were deposited onto freshly cleaved mica substrates, and amplitude images and 3D topographic maps were acquired under ambient conditions (25°C, 50% RH) with a silicon probe (RTESPA-300). Average roughness (Ra) and root mean square roughness (Rq) were quantified from height images using the following definitions:

$Ra=\frac{1}{N}\sum_{i=1}^{N} \left| z_{i}-\bar{z} \right|$ Equation 1

$Rq=\sqrt{\frac{1}{N}\sum_{i=1}^{N} \left( z_{i}-\bar{z} \right)^{2}}$ Equation 2

Where N was the number of data points, z_i_ ​was the height of the ith data point, $\bar{z}$ was the average height of data points.

Local Young’s modulus was determined through force-distance curve measurements using the Hertz contact model:

$F=\frac{4}{3}\times E\times R^{\frac{1}{2}}\times\delta^{\frac{3}{2}}$ Equation 3

Where F was the force applied to the fibers, E was Young’s modulus of the fiber, R was the radius of the AFM probe, δ was the surface deformation of the fibers.

*Physicochemical Characterization of Fibers:* Polarized optical microscopy was used to examine the molecular orientation of the as-spun and post-stretched fibers. Ori fibers were stretched approximately threefold and C1 fibers twofold prior to imaging. Images were acquired using a Nikon ECLIPSE LV100N POL microscope. FTIR spectra of Ori and C1 fiber bundles were collected using PerkinElmer FTIR spectrometer (Waltham, MA, USA) in the region of 4000-400 cm^-1^. The secondary-structure contents were determined through spectral deconvolution of the amide I region (1700-1600 cm^-1^) using PeakFit (Origin 9.0 software), with a full width at half maximum (FWHM) of 20–25 cm^-1^ to achieve the best agreement with the experimental spectra. Fiber samples for SAXS and Raman measurements were prepared by aligning the microfluidic-spun fibers in parallel and assembling them into bundles approximately 3 mm in width and 5 cm in length. Small-angle X-ray scattering (SAXS) experiments were conducted at an X-ray energy of 10.0 keV, corresponding to a wavelength of λ = 1.5406 Å. Measurements on C1 fibers encompassed the scattering vector q range from 0.1 to 4 nm^-1^, (q is the scattering vector, q = 4π/(λ sinθ), where 2θ represents the scattering angle. Polarized Raman spectra were acquired using an HR Evolution Raman spectrometer (HORIBA Scientific, Japan) with a 514 nm laser and a 1200 lines/mm grating in the spectral range of 4000-400 cm^-1^. Ori and C1 fiber bundles were oriented along the x-axis and y-axis, respectively, with data collected at three different positions every 10 seconds, accumulating 16 scans each time.

*Molecular Dynamics Simulations**:* The primary structures of the Ori and C1 spidroins were determined by their amino acid sequence, with C1 containing cysteine substitutions at the terminal poly-alanine region. Monomers were initially placed in a 3×5 lattice with anti-parallel stacking along the hydrogen bonding direction and parallel stacking in the side chain direction.^[3]^ The spacing between monomers was set to 5 Å in both directions. The assembled units were solvated with explicit water corresponding to 20% hydration. All atomic interactions were described using the GROMOS54a7 force field,^[4]^ and water molecules were modeled using the SPC model. After energy minimization, each system was equilibrated under the NVT ensemble for 200 ns at 300 K. The temperature was increased gradually from 0 to 300 K using simulated annealing. A timestep of 2 fs was used, and trajectory frames were saved every 10 ps. Periodic boundary conditions were applied with at least 20 Å of padding around the fiber to prevent image interactions. To identify potential disulfide linkages, the C1 model without predefined disulfide bonds was first equilibrated. The resulting structures were analyzed using the MODIP algorithm, applying cutoffs of 7 Å for Cα–Cα and 6 Å for Cβ–Cβ distances. Based on these criteria, 20 disulfide bonds were predicted to form among cysteine residues in the C1 assembly.

*Shear Deformation Simulations:* Steered molecular dynamics was used to evaluate the mechanical response of the β-sheet crystals. One end of a monomer was pulled along the x-direction at a velocity of 0.01 nm ps^-1^ with a spring constant of 1000 kJ mol^-1^ nm^-2^, mimicking the lateral shear stresses experienced in native silk fibers. Each monomer was pulled separately, yielding 15 simulations for both Ori and C1. Representative snapshots illustrate the deformation of the central chain and the presence or rupture of disulfide bonds at polyA sites. Force–extension curves were obtained by tracking the applied force and the displacement of the pulled atoms. Secondary-structure evolution was analyzed by extracting DSSP assignments from the trajectories. For each residue, the fraction of simulation time spent in β-sheet conformation was calculated and visualized as a heat map using Origin 9.0. All the simulations were carried out using GROMACS 2021.4 package, the visualizations were performed through VMD, and the analyses of secondary structure was through the STRIDE algorithm.^[5]^

*Statistical Analysis:* Numerical data were presented as mean ± standard deviation (SD). To assess statistical significance, one-way analysis of variance (ANOVA) was performed with Origin 9.0. Each experiment was conducted in quadruplicate, and significance levels were denoted as follows: *P < 0.05, **P < 0.01, ***P < 0.001.

**Supplementary Figures and Tables**

**Figure S1.** Computational screening of steric zipper–forming potential in wild-type and cysteine-modified hexapeptides.

**Figure S2.** Expression and purification of recombinant spidroin variants.

**Figure S3.** SEC–MALS analysis of recombinant spidroin variants.

**Figure S4.** SDS–PAGE analysis of recombinant spidroin variants under reducing and oxidizing conditions.

**Figure S5.** Thermal stability of recombinant spidroins.

**Figure S6.** Size evolution of protein droplets during LLPS.

**Figure S7.** Assembly kinetics and thermal response of spidroin variants.

**Figure S8.** Mesoscale morphology of C1-derived fibers.

**Figure S9.** Beads-on-a-string morphology during LLPS.

**Figure S10.** Molecular alignment in as-spun and stretched fibers.

**Figure S11.** Mechanical profiles of control and modified fibers.

**Figure S12.** Mechanical characteristics under different redox conditions.

**Figure S13.** FTIR analysis of secondary structures in silk fibers.

**Figure S14.** Illustration of the initial MD structures of C1 (a) and Ori (b).

**Figure S15.** Molecular dynamics simulation of mechanical responses.

**Figure S16.** Stability of the β sheet domains under equilibrium conditions without pulling.

**Figure S17.** Surface morphology of silk fibers revealed by AFM.

**Figure S18.** Polarized Raman spectroscopy of aligned fibers.

**Table S1.** Amino acid sequences of original (Ori) and engineered constructs (C1, C3, C5).

**Table S2.** Tensile mechanical properties of native spider silks and other recombinant spidroin fibers.

**Table S3.** Mechanical properties of Ori fibers and C1 fibers.


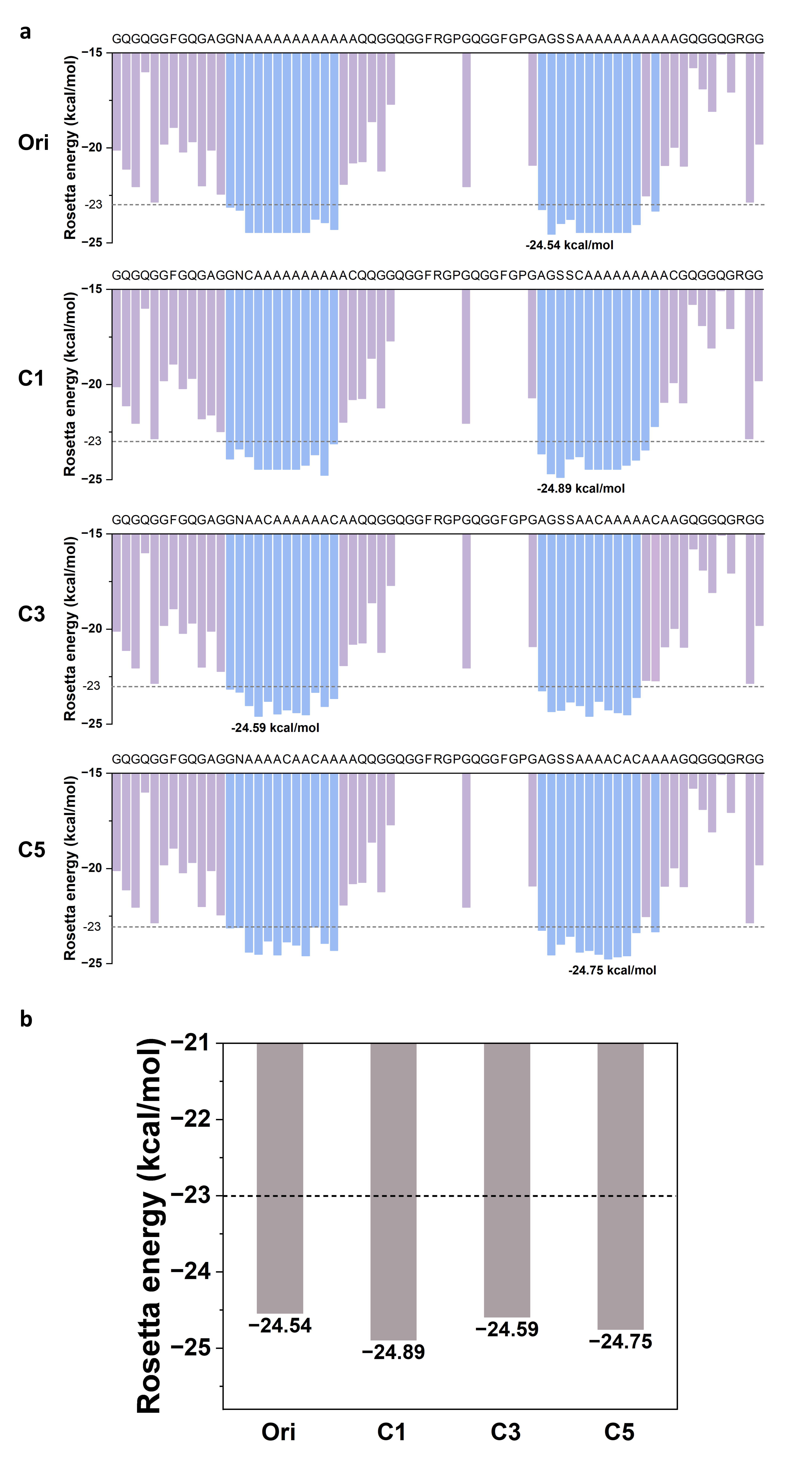


Figure S1. Computational screening of steric zipper–forming potential in wild-type and cysteine-modified hexapeptides. (a) Distribution of Rosetta energy scores for all predicted hexapeptides. Blue bars denote sequences with energies ≤ –23 kcal/mol (threshold for likely steric zipper formation), while purple bars represent higher-energy sequences unlikely to form ordered β-sheet structures. (b) The lowest-energy conformations identified for Ori and engineered variants (C1, C3, and C5), highlighting the energetic advantage introduced by terminal cysteine substitutions.


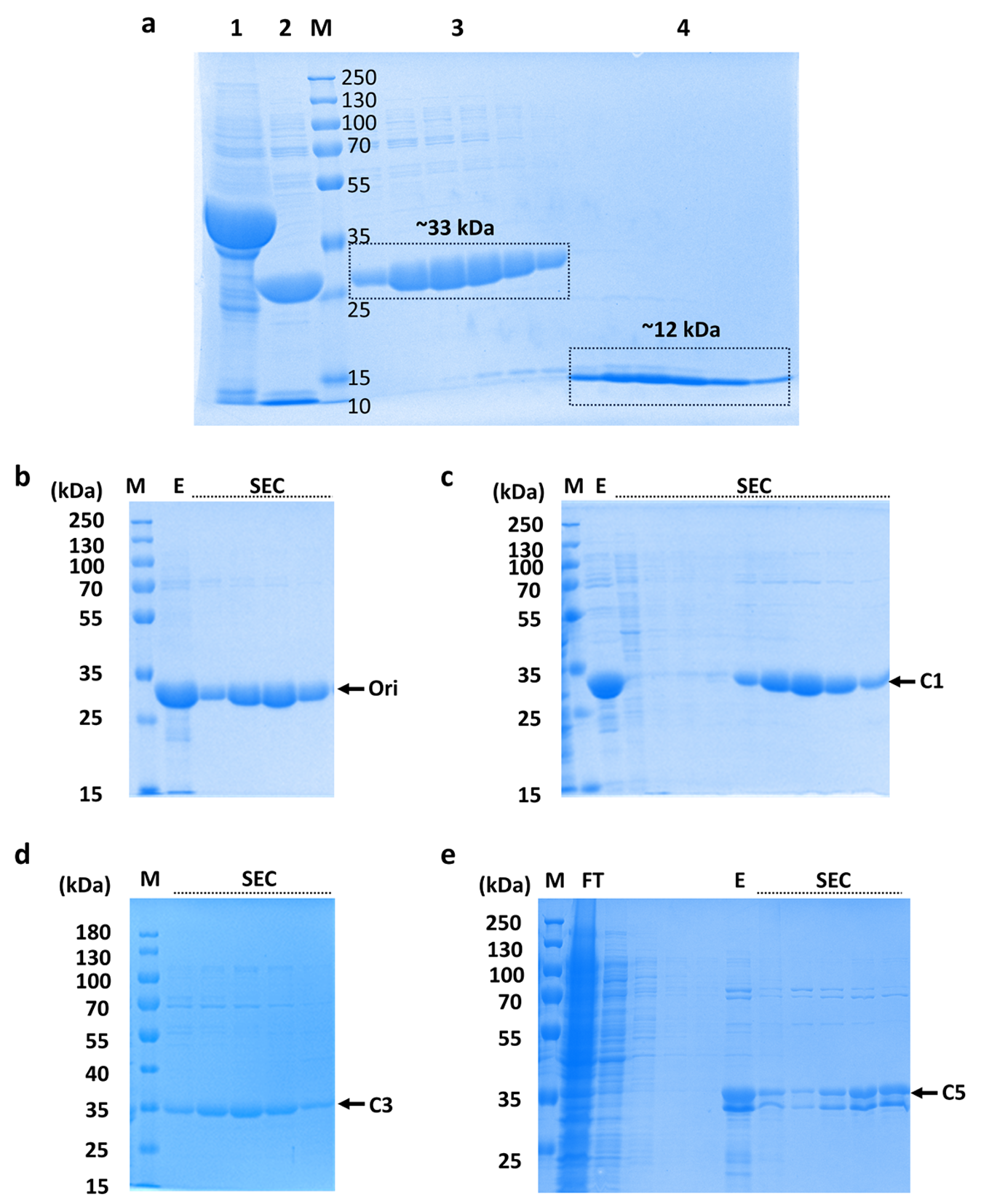


**Figure S2. Expression and purification of recombinant spidroin variants. (a)** SDS–PAGE analysis showing the TrxA-fusion protein before and after HRV 3C cleavage. Lane 1: fusion protein before cleavage (~45 kDa); Lane 2: after HRV 3C digestion; Lane 3: cleaved target (~33 kDa); Lane 4: free TrxA (~12 kDa); M: protein marker. SDS–PAGE analyses of different purification fractions for **(b)** Ori, **(c)** C1, **(d)** C3, and **(e)** C5. FT: flow-through fraction; E: Ni–NTA elution; SEC: size-exclusion chromatography elution.

**
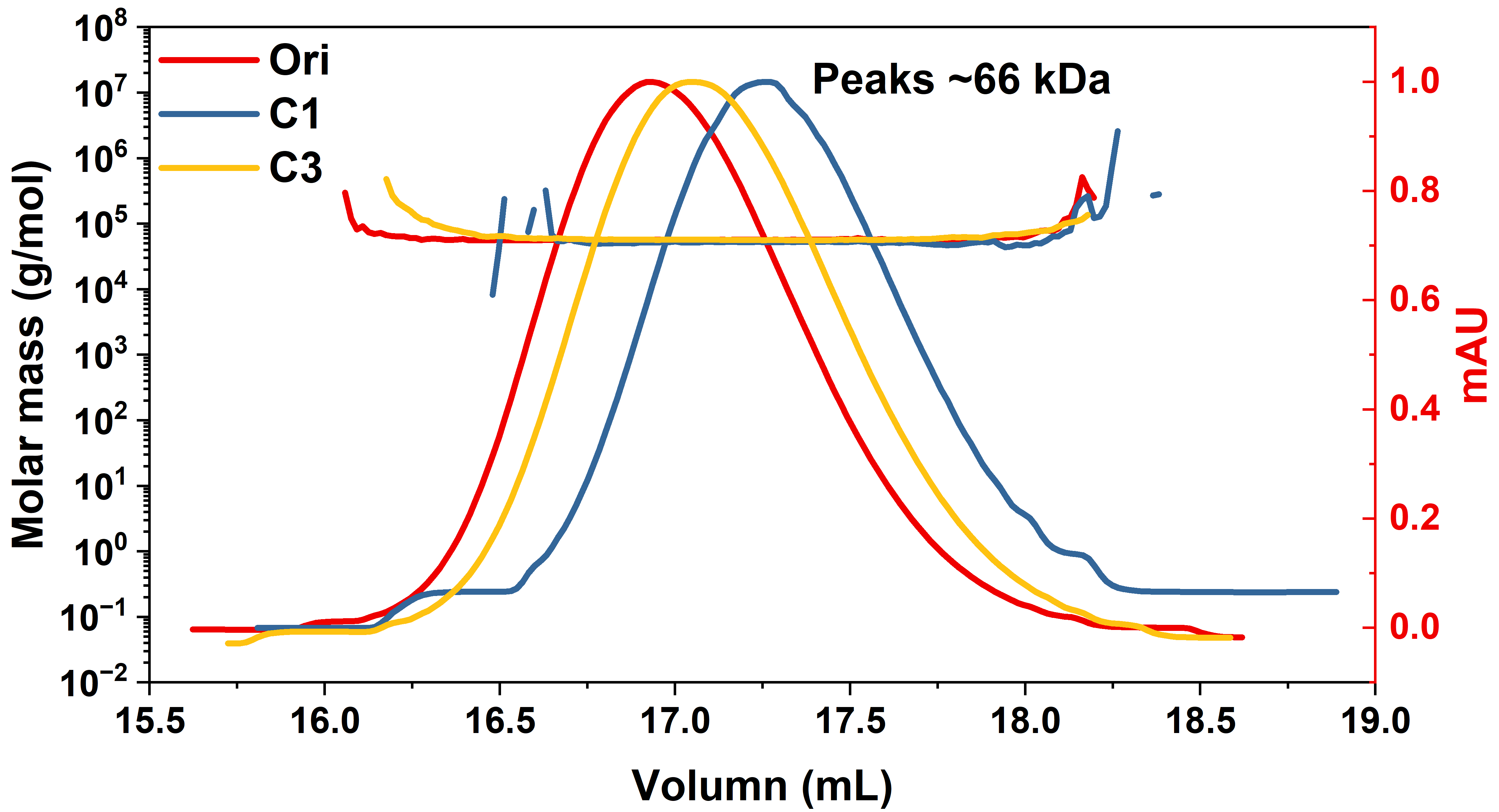
**

**Figure S3. SEC–MALS analysis of recombinant spidroin variants.** Ori, C1, and C3 elute predominantly as dimers with an apparent molecular weight of ~66 kDa under the measurement conditions (50 mM Tris–HCl pH 7.5, 100 mM NaCl, 1 mM EDTA, 1 mM DTT).


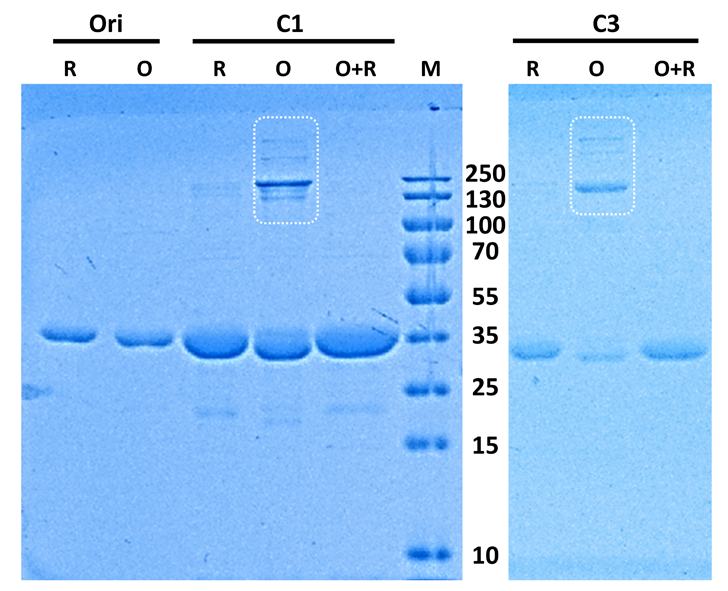


**Figure S4. SDS–PAGE analysis of recombinant spidroin variants under reducing and oxidizing conditions.** “R” indicates reducing conditions, “O” indicates oxidizing conditions, and “O+R” denotes oxidized samples subsequently reduced with DTT. “M” represents the protein marker.


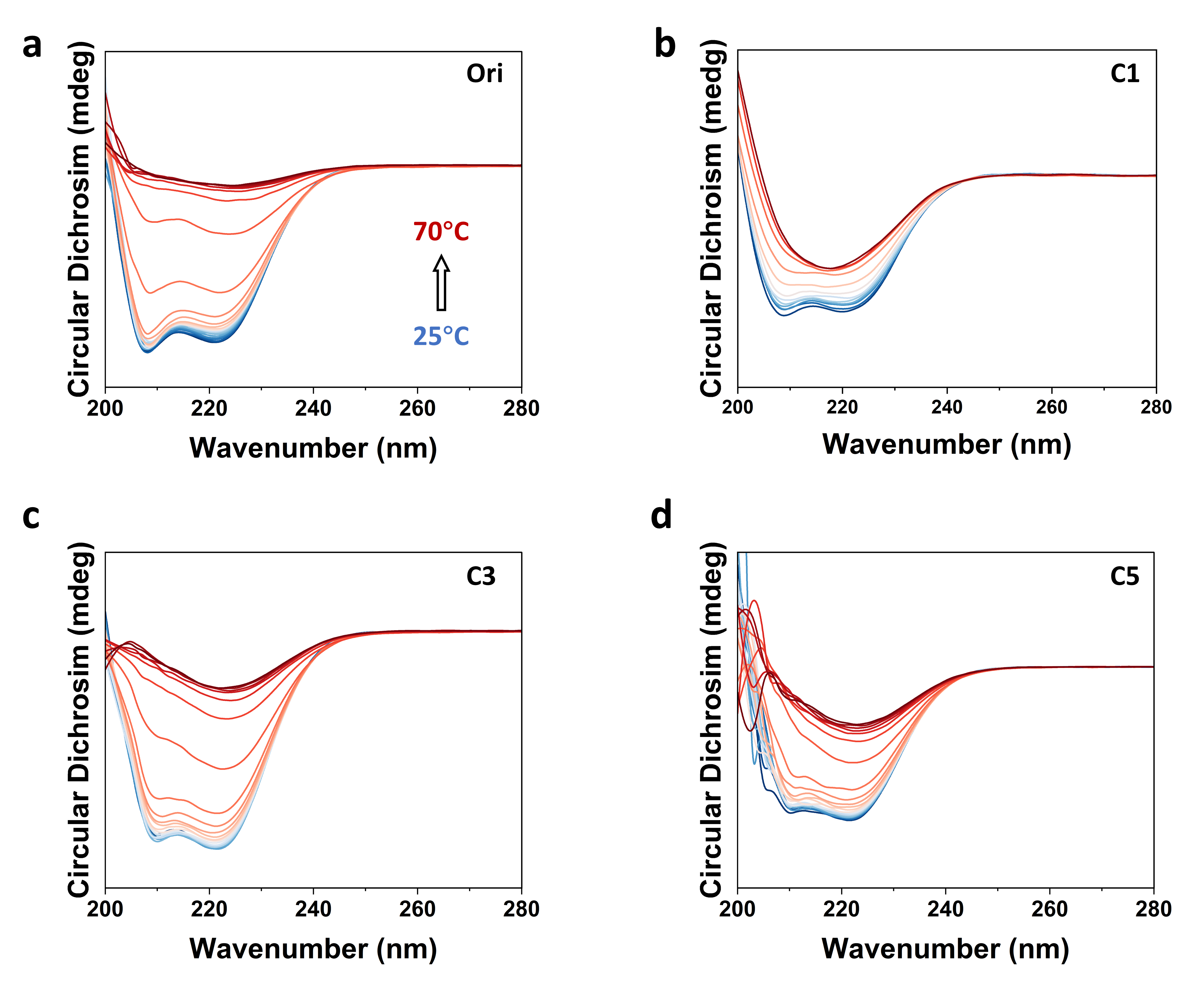


Figure S5. Thermal stability of recombinant spidroins. Circular dichroism spectra of (a) Ori, (b) C1, (c) C3, and (d) C5 recorded from 25 °C to 70 °C, indicating secondary structure evolution upon heating.


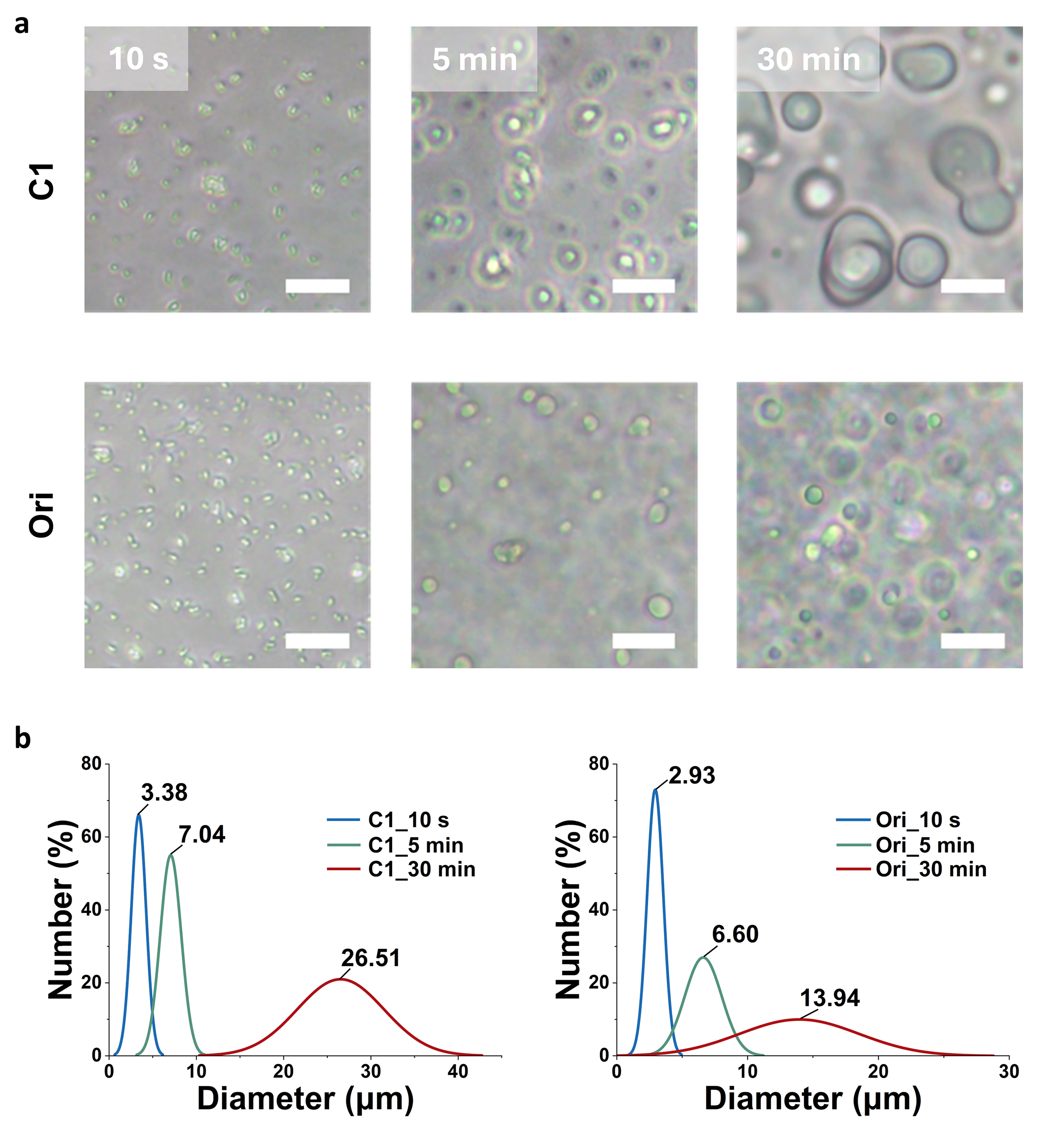


Figure S6. Size evolution of protein droplets during LLPS. (a) Optical microscopy images of Ori and C1 droplets after incubation for 5 and 30 min (scale bar: 20 μm). (b) Quantification of average droplet diameters at 10 s, 5 min, and 30 min time points.


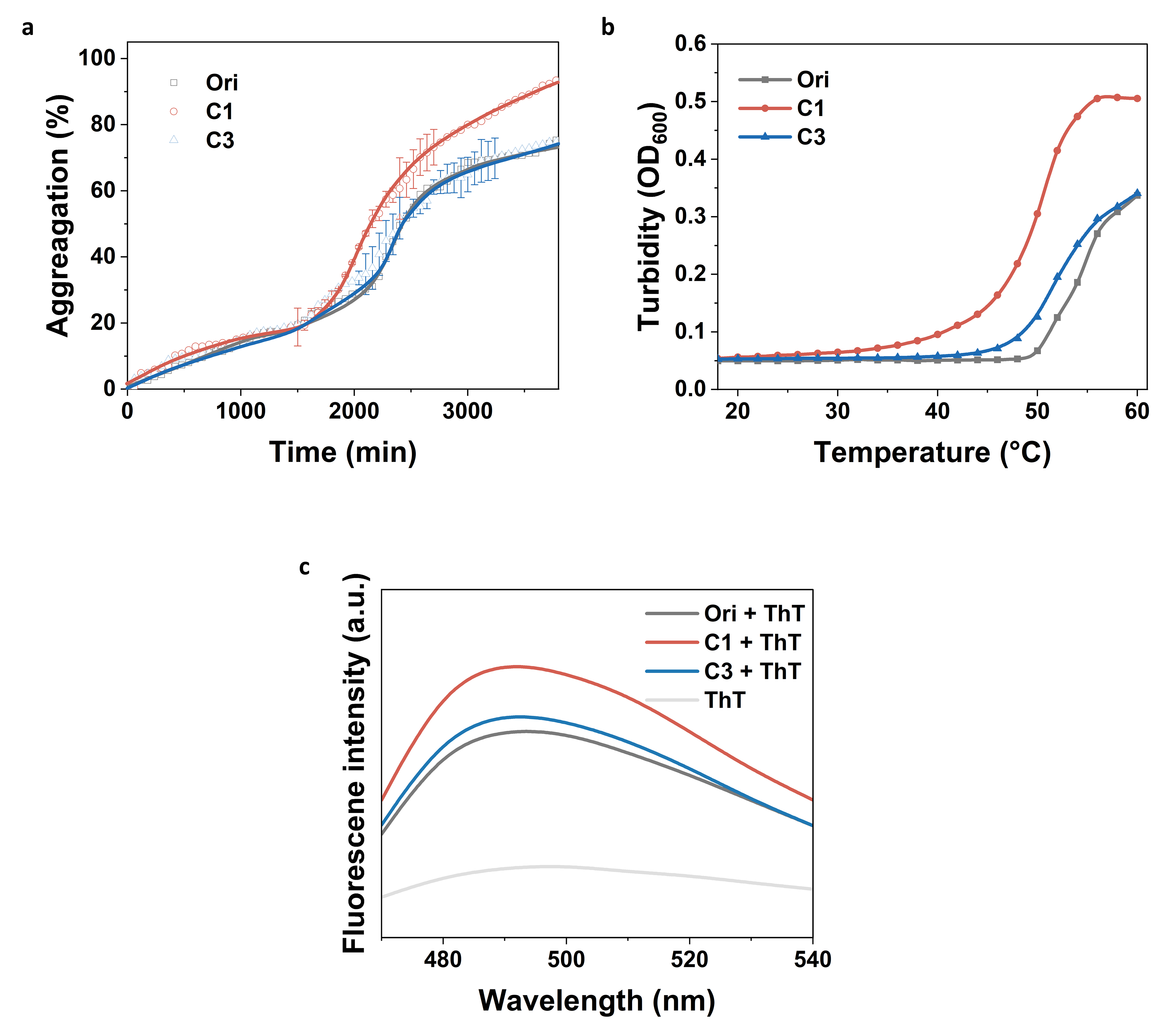


Figure S7. Assembly kinetics and thermal response of spidroin variants. (a) Fibril assembly kinetics of Ori, C1, and C3 in phosphate buffer (pH 8.0) monitored by optical density at 340 nm. (b) Temperature-dependent self-assembly behavior of samples in 0.3 M potassium phosphate buffer, assessed by OD_600_ from 18–60 °C. (c) Thioflavin T fluorescence spectra of Ori, C1, and C3 at equimolar concentrations.


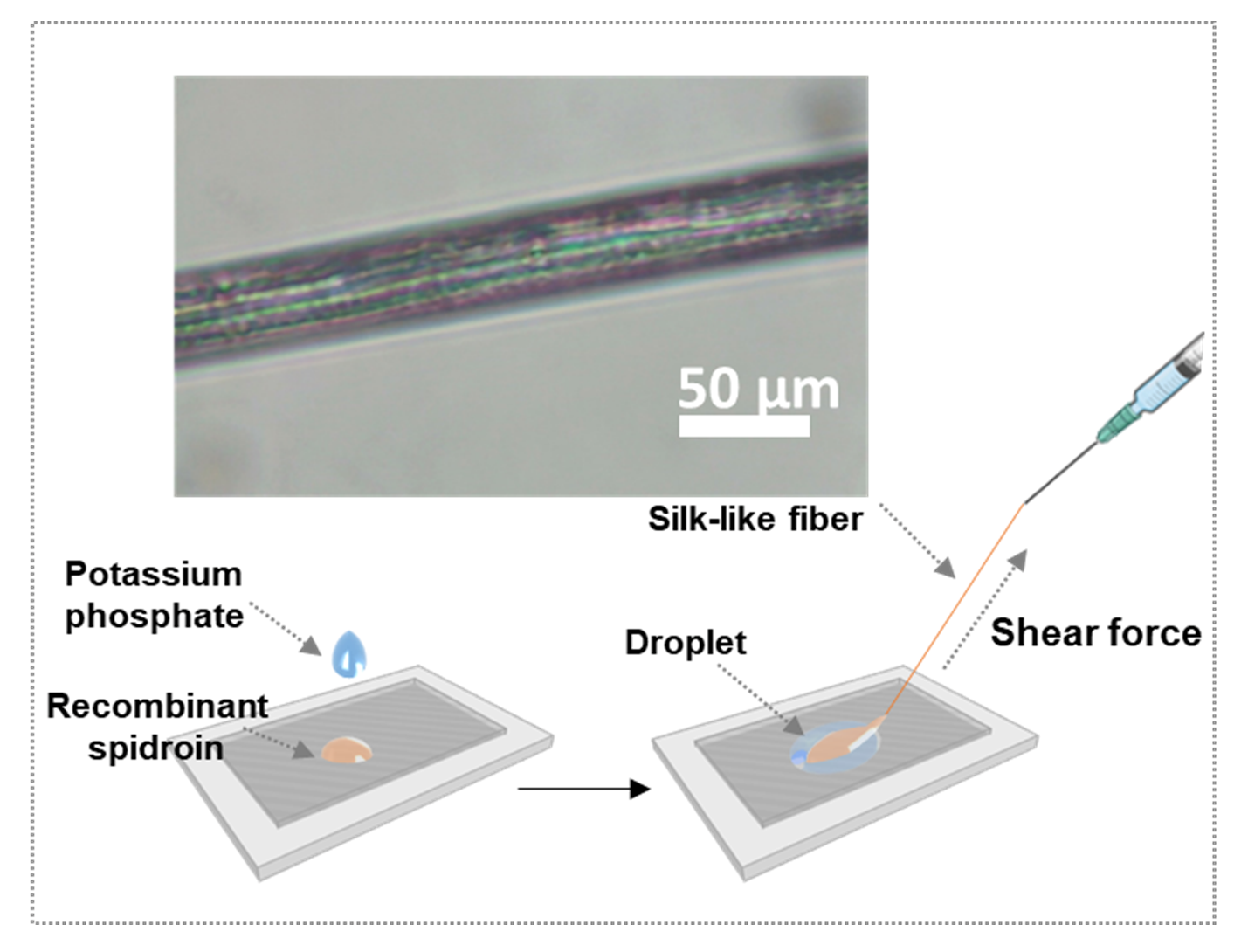


Figure S8. Mesoscale morphology of C1-derived fibers. Optical microscopy images showing the fibrous structures formed from 0.5 μL of 100 mg mL^-1^ C1 protein following the protocol described in the Methods. Scale bars are indicated on the images.


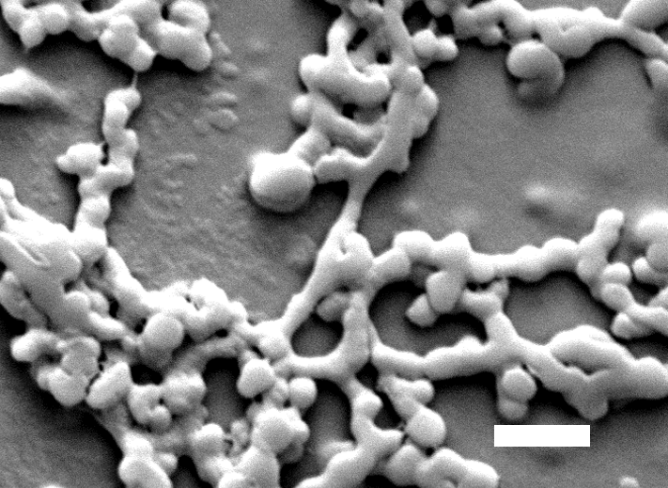


Figure S9. Beads-on-a-string morphology during LLPS. SEM image of intermediate structures formed during C1 LLPS, exhibiting a "beads-on-a-string" morphology. Scale bar: 5 μm.


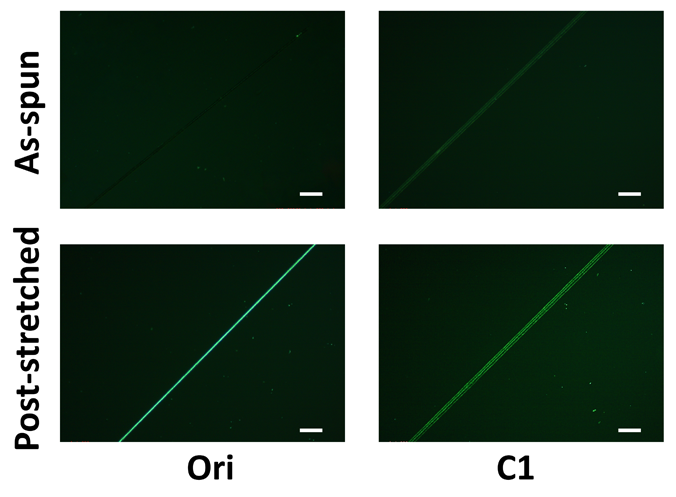


Figure S10. Molecular alignment in as-spun and stretched fibers. Polarized optical microscopy images of as-spun and post-stretched fibers from Ori and C1 (scale bar: 50 μm). As-spun C1 exhibits distinct birefringence, suggesting intrinsic molecular alignment. Ori fibers were stretched 3×, and C1 fibers 2× before mechanical testing.


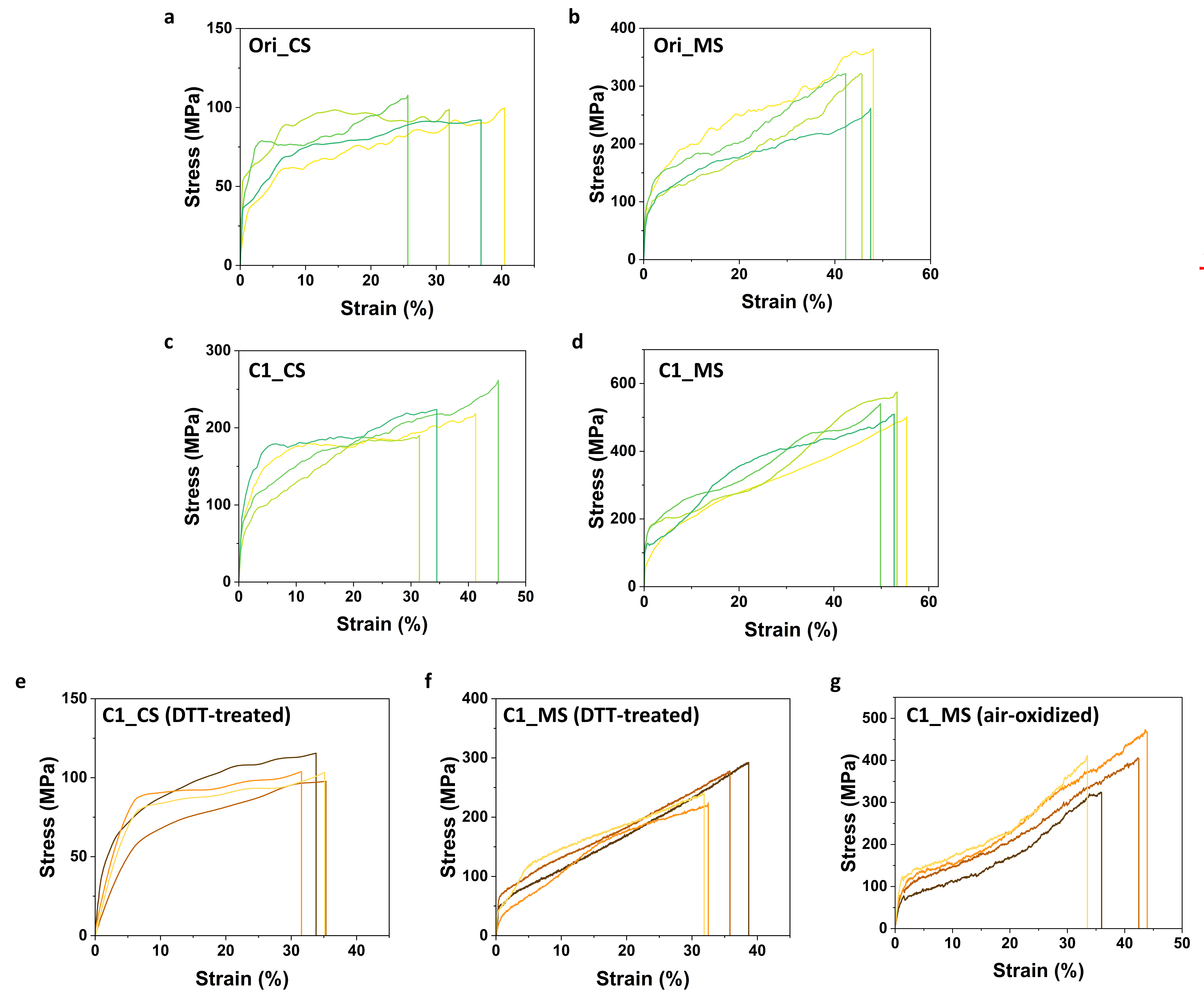


Figure S11. Mechanical profiles of control and modified fibers. Representative stress–strain curves of (a) Ori_CS, (b) Ori_MS, (c) C1_CS, (d) C1_MS, (e) C1_CS (DTT-treated), (f) C1_MS (DTT-treated), (g) C1_MS (air-oxidized) fibers. Data were collected from four replicates per group (n = 4).


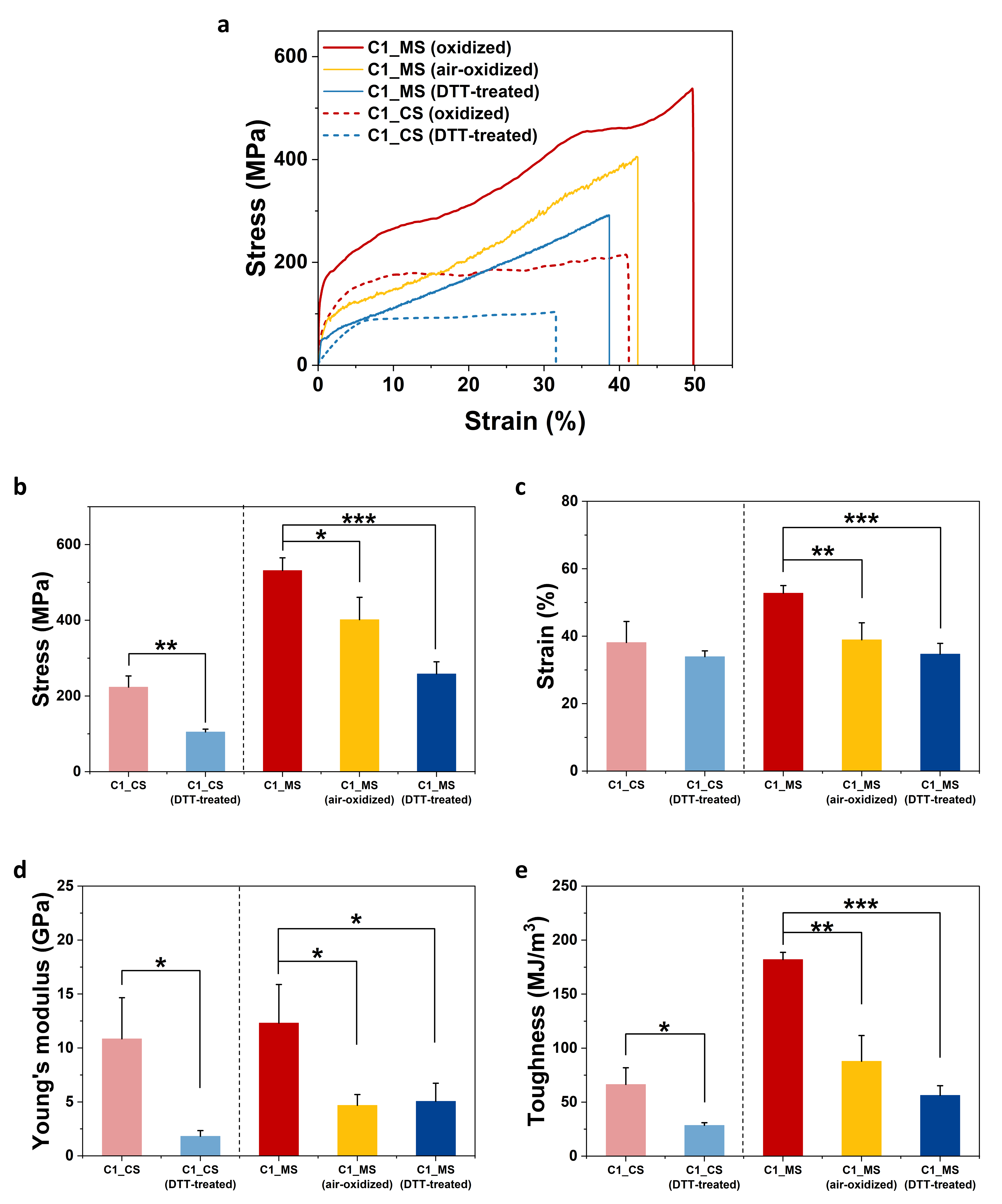


Figure S12. Mechanical characteristics under different redox conditions. (a) Representative stress–strain curves of fibers subjected to reducing or oxidizing treatments. (b) Tensile strength of the corresponding fiber groups. (c) Strain at break. (d) Young’s modulus. (e) Toughness. Data were collected from four replicates per group (n = 4).


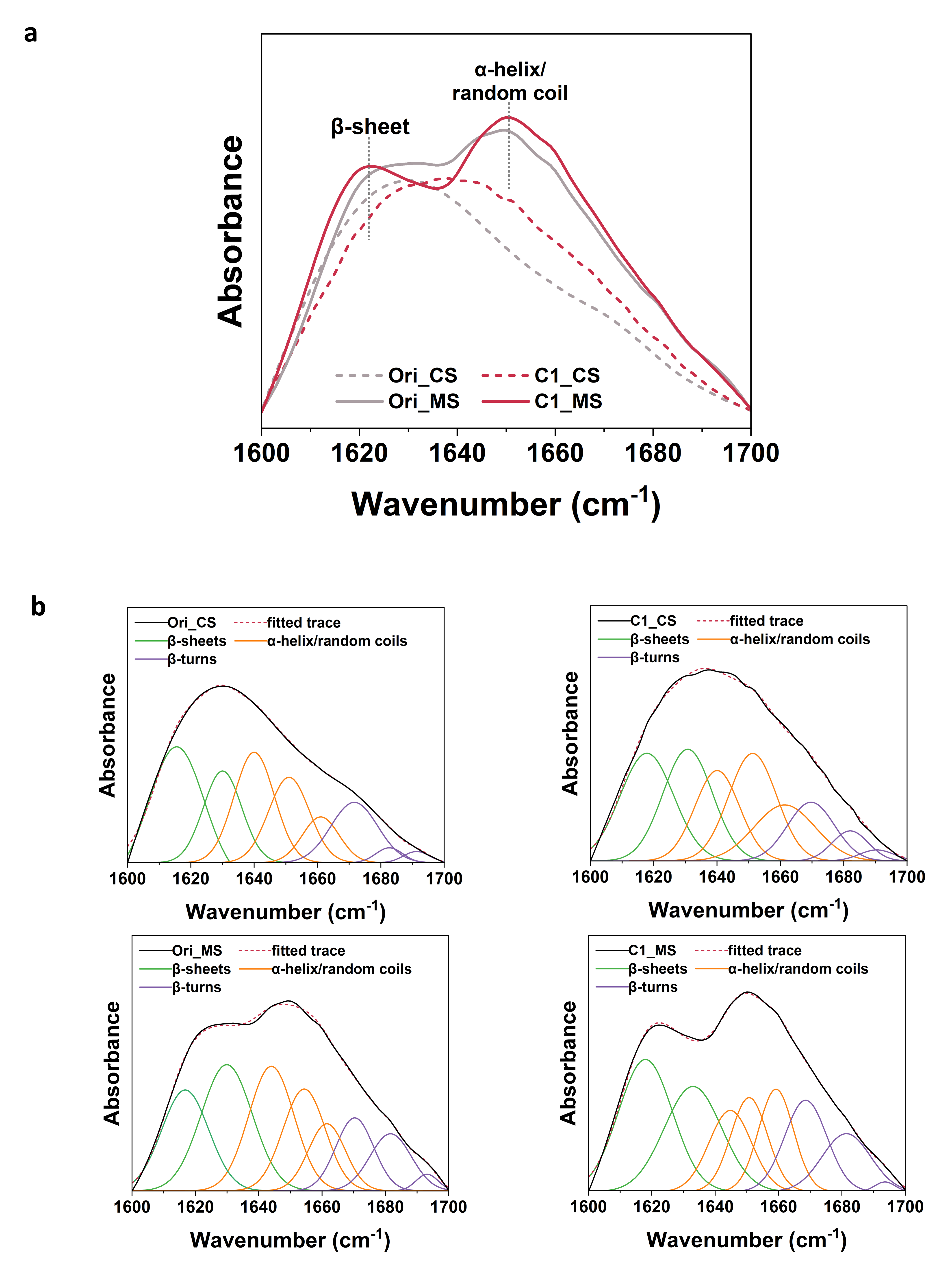


Figure S13. FTIR analysis of secondary structures in silk fibers. (a) FTIR spectra of Ori, C1, C3, and C5 fibers in the amide I region (1600–1700 cm^-1^). Peaks near 1633 cm^-1^ and 1645 cm^-1^ correspond to β-sheet and α-helix/random coil structures, respectively. (b) Quantification of secondary structure content based on peak deconvolution.


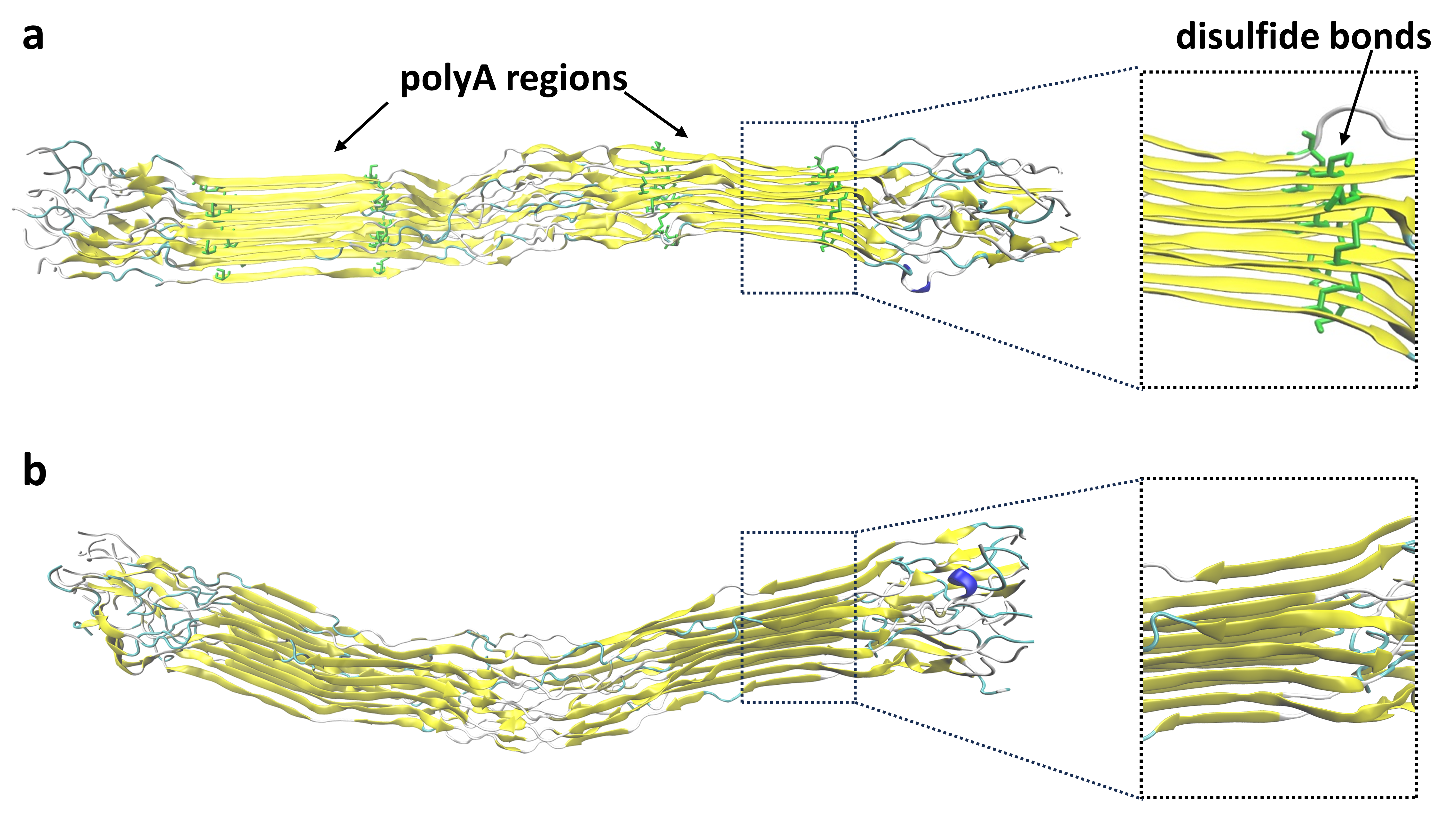


**Figure S14. Illustration of the initial MD structures of C1** **(a) and Ori (b).** The enlarged view on the right highlights the parallel β-strands and the disulfide bonds in C1. The secondary structures are depicted in New Cartoon style, with β-sheets, β-turns, random coils, and α-helices shown in yellow, cyan, white, and blue, respectively, and cysteines forming disulfide bonds shown in green.


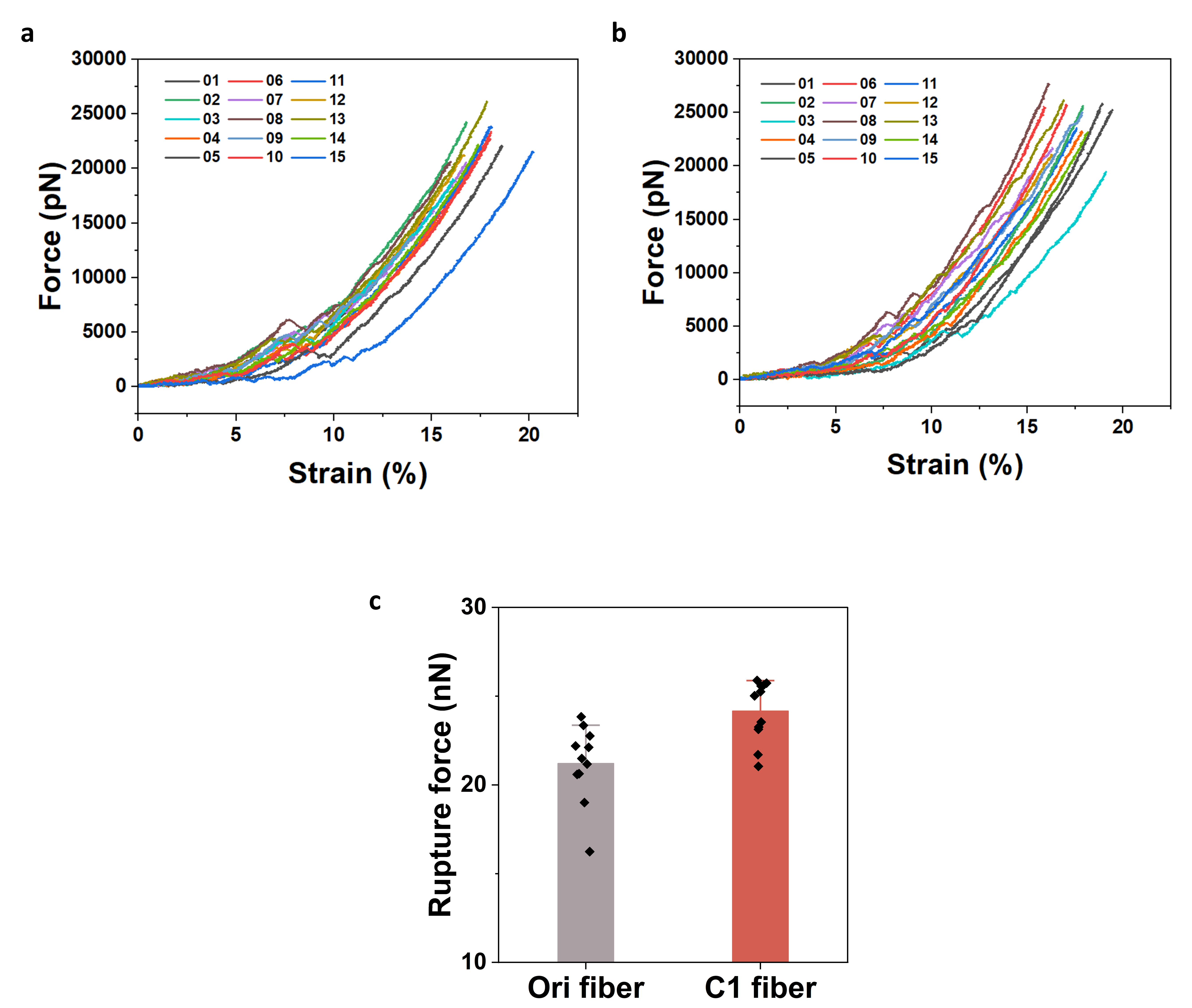


Figure S15. Molecular dynamics simulation of mechanical responses. (a–b) Force–displacement curves of Ori (a) and C1 (b) fibers collected over 15 independent pulling simulations. (c) Statistical comparison of peak rupture force between variants. Error bars indicate standard deviation.


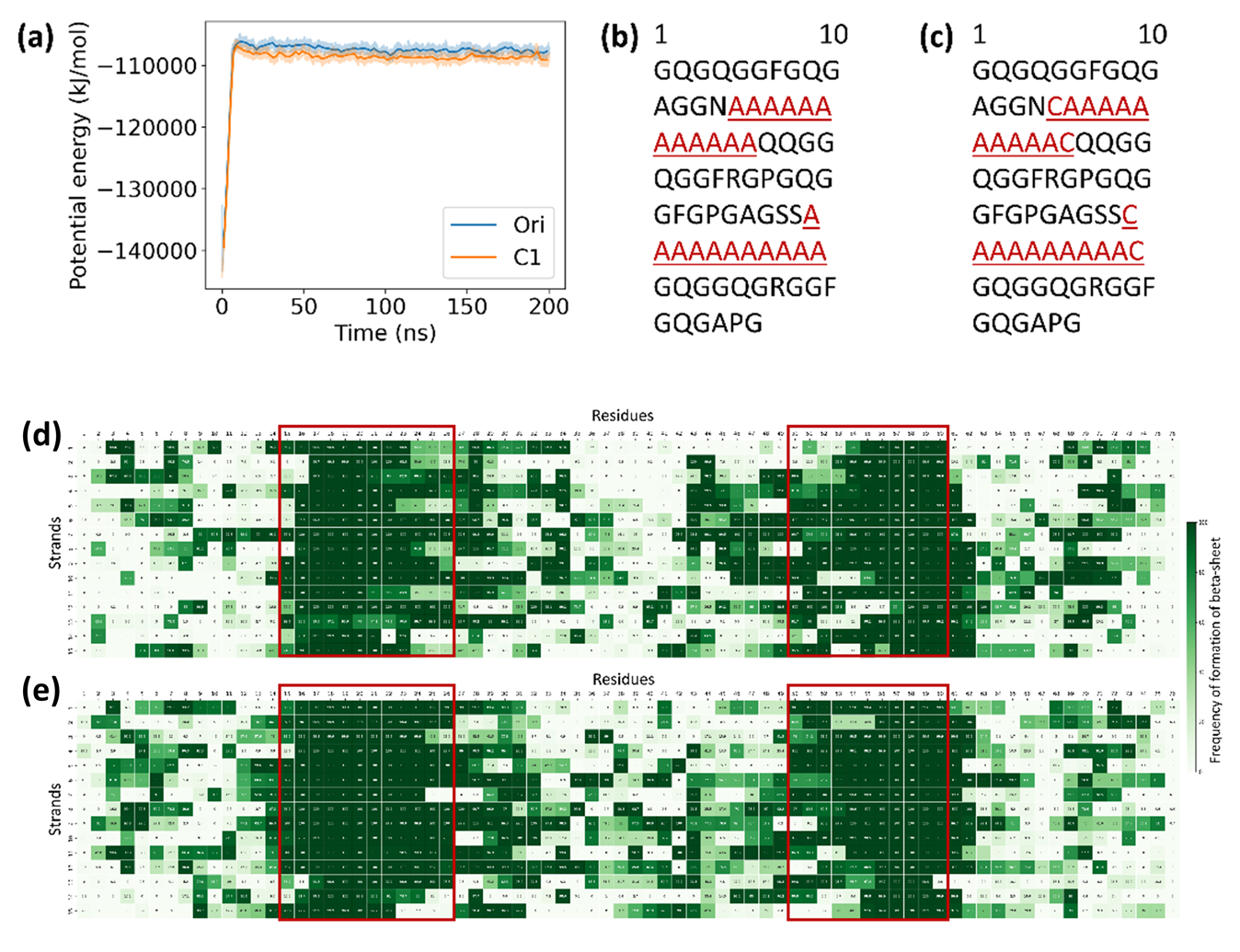


**Figure S16. Stability of the β sheet domains under equilibrium conditions without pulling. (a)** Potential energy profile during the equilibrium simulation. **(b)** Sequence of the Ori fiber and **(c)** sequence of the C1 fiber, where the underlined red residues correspond to the β sheet forming regions. **(d)** Heatmap of the β sheet formation frequency at each residue position in the Ori fiber and **(e)** in the C1 fiber. The red boxes indicate the β sheet domains.


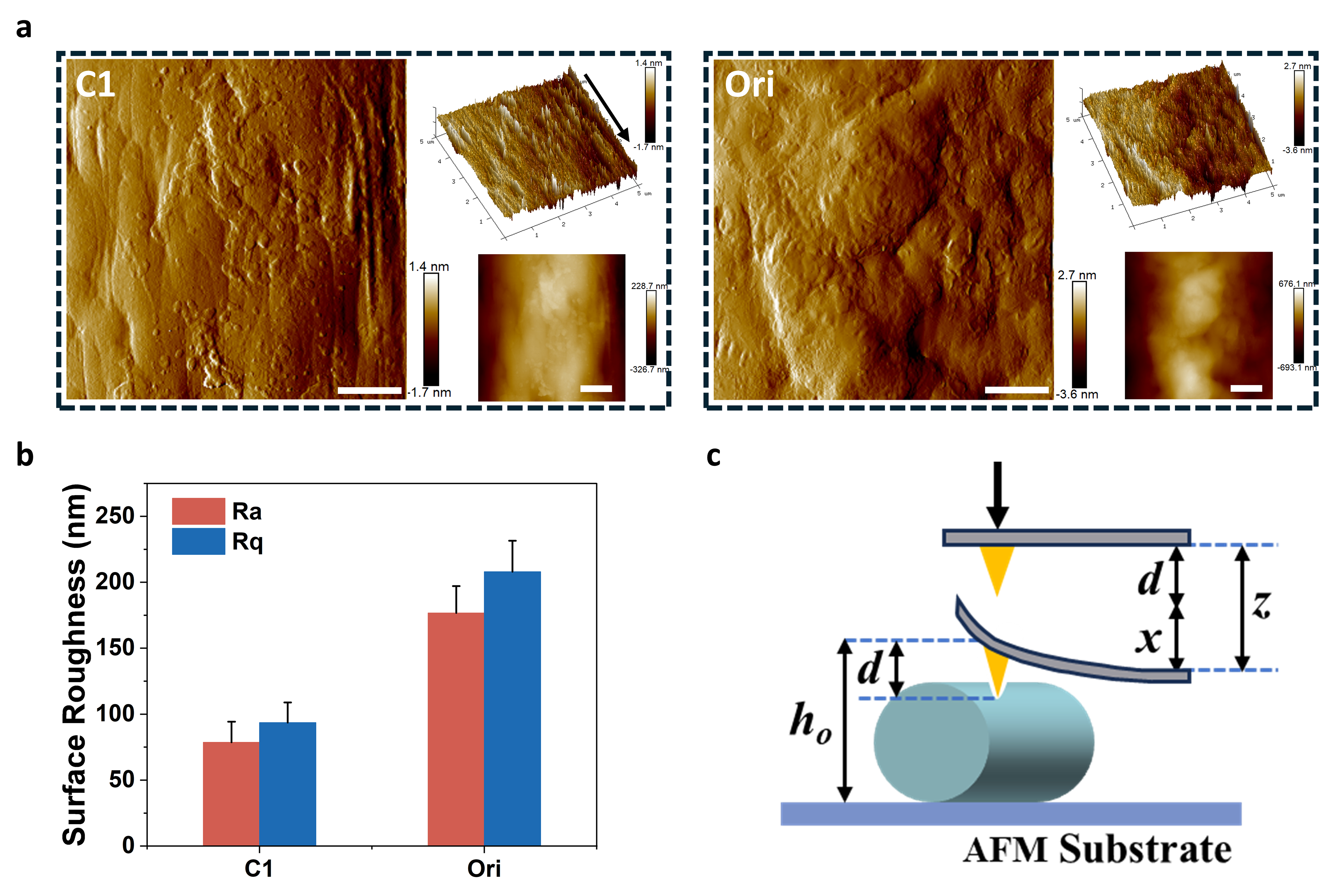


Figure S17. Surface morphology of silk fibers revealed by AFM. (a) AFM amplitude images (left) and corresponding 3D surface reconstructions (right) for Ori and C1 fibers. (b) Surface roughness values (Ra and Rq) are reported as mean ± SD (n = 3). (c) A schematic diagram illustrates the tapping mode principle used in AFM analysis.


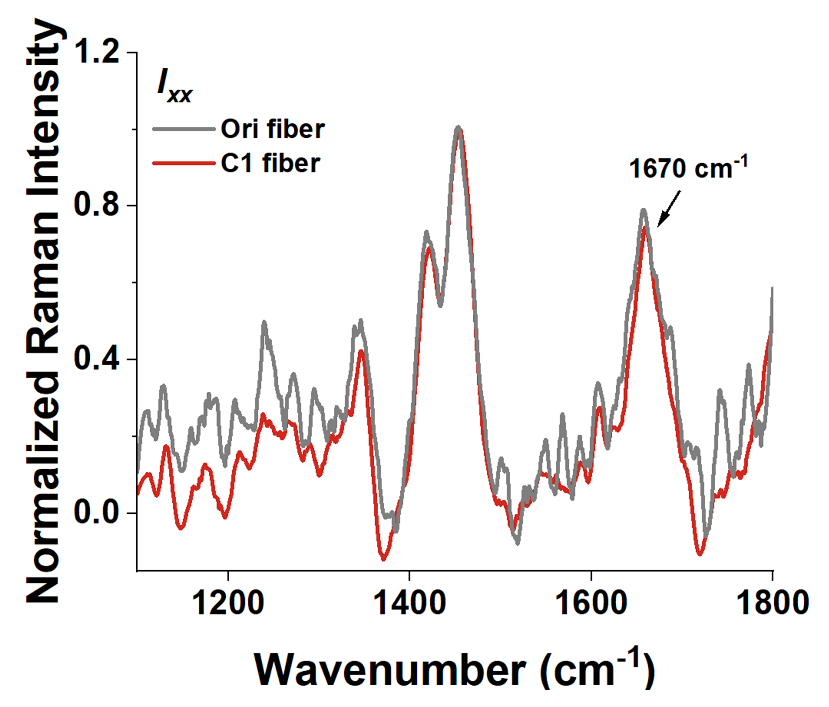


Figure S18. Polarized Raman spectroscopy of aligned fibers. Polarized Raman spectra of Ori and C1 fibers measured in the I_xx_ configuration, highlighting β-sheet alignment along the fiber axis.

Table S1. Amino acid sequences of original (Ori) and engineered constructs (C1, C3, C5) thereof:

| **Name** | **Amino acid sequence** |
| --- | --- |
| Ori protein | MSDKIIHLTDDSFDTDVLKADGAILVDFWAEWCGPCKMIAPILDEIADEYQGKLTVAKLNIDQNPGTAPKYGIRGIPTLLLFKNGEVAATKVGALSKGQLKEFLDANLAGSGSGHMHHHHHHSSGLEVLFQGPGSSHTTPWTNPGLAENFMNSFMQGLSSMPGFTASQLDDMSTIAQSMVQSIQSLAAQGRTSPNKLQALNMAFASSMAEIAASEEGGGSLSTKTSSIASAMSNAFLQTTGVVNQPFINEITQLVSMFAQAGMNDVSAGQGQGGFGQGAGGNAAAAAAAAAAAAQQGGQGGFRGPGQGGFGPGAGSSAAAAAAAAAAAGQGGQGRGGFGQGAPGVTSGGYGYGTSAAAGAGVAAGSYAGAVNRLSSAEAASRVSSNIAAIASGGASALPSVISNIYSGVVASGVSSNEALIQALLELLSALVHVLSSASIGNVSSVGVDSTLNVVQDSVGQYVG* |
| C1 protein | MSDKIIHLTDDSFDTDVLKADGAILVDFWAEWCGPCKMIAPILDEIADEYQGKLTVAKLNIDQNPGTAPKYGIRGIPTLLLFKNGEVAATKVGALSKGQLKEFLDANLAGSGSGHMHHHHHHSSGLEVLFQGPGSSHTTPWTNPGLAENFMNSFMQGLSSMPGFTASQLDDMSTIAQSMVQSIQSLAAQGRTSPNKLQALNMAFASSMAEIAASEEGGGSLSTKTSSIASAMSNAFLQTTGVVNQPFINEITQLVSMFAQAGMNDVSAGQGQGGFGQGAGGNCAAAAAAAAAACQQGGQGGFRGPGQGGFGPGAGSSCAAAAAAAAACGQGGQGRGGFGQGAPGVTSGGYGYGTSAAAGAGVAAGSYAGAVNRLSSAEAASRVSSNIAAIASGGASALPSVISNIYSGVVASGVSSNEALIQALLELLSALVHVLSSASIGNVSSVGVDSTLNVVQDSVGQYVG* |
| C3 protein | MSDKIIHLTDDSFDTDVLKADGAILVDFWAEWCGPCKMIAPILDEIADEYQGKLTVAKLNIDQNPGTAPKYGIRGIPTLLLFKNGEVAATKVGALSKGQLKEFLDANLAGSGSGHMHHHHHHSSGLEVLFQGPGSSHTTPWTNPGLAENFMNSFMQGLSSMPGFTASQLDDMSTIAQSMVQSIQSLAAQGRTSPNKLQALNMAFASSMAEIAASEEGGGSLSTKTSSIASAMSNAFLQTTGVVNQPFINEITQLVSMFAQAGMNDVSAGQGQGGFGQGAGGNAACAAAAAACAAQQGGQGGFRGPGQGGFGPGAGSSAACAAAAACAAGQGGQGRGGFGQGAPGVTSGGYGYGTSAAAGAGVAAGSYAGAVNRLSSAEAASRVSSNIAAIASGGASALPSVISNIYSGVVASGVSSNEALIQALLELLSALVHVLSSASIGNVSSVGVDSTLNVVQDSVGQYVG* |
| C5 protein | MSDKIIHLTDDSFDTDVLKADGAILVDFWAEWCGPCKMIAPILDEIADEYQGKLTVAKLNIDQNPGTAPKYGIRGIPTLLLFKNGEVAATKVGALSKGQLKEFLDANLAGSGSGHMHHHHHHSSGLEVLFQGPGSSHTTPWTNPGLAENFMNSFMQGLSSMPGFTASQLDDMSTIAQSMVQSIQSLAAQGRTSPNKLQALNMAFASSMAEIAASEEGGGSLSTKTSSIASAMSNAFLQTTGVVNQPFINEITQLVSMFAQAGMNDVSAGQGQGGFGQGAGGNAAAACAACAAAAQQGGQGGFRGPGQGGFGPGAGSSAAAACACAAAAGQGGQGRGGFGQGAPGVTSGGYGYGTSAAAGAGVAAGSYAGAVNRLSSAEAASRVSSNIAAIASGGASALPSVISNIYSGVVASGVSSNEALIQALLELLSALVHVLSSASIGNVSSVGVDSTLNVVQDSVGQYVG* |

Table S2. Tensile mechanical properties of native spider silks and other recombinant spidroin fibers.

| **Materials** | **Molecular**  **weight**  **(kDa)** | **Preserving native structure** | **Strength**  **(MPa)** | | **Elongation (%)** | **Toughness (MJ/m^3^)** | | **Ref** | |
| --- | --- | --- | --- | --- | --- | --- | --- | --- | --- |
| **C1_CS fibers** | **33** | **+** | **223.62±29.38** | **38.11±6.26** | | | **66.44± 5.43** | | **This**  **work** |
| **C1_MS fibers** | **33** | **+** | **531.76±33.46** | **52.78±2.27** | | | **182.06±6.67** | |  |
| NTD-eADF3-CTD | 75 | - | 239 ± 12 | 59 ± 1 | | | 71 ± 3 | | ^[6]^ |
| heterodimer of NTD-eADF3/4-CTD | 150 | + | 834 ± 34 | 32 ± 1 | | | 143 ± 6 | |  |
| (A_3_I)_3_-A_14_ | 33 | + | 131 | 48±55.8 | | | 18.2±20.3 | | ^[7]^ |
| 16×spidroin | 44 | - | 82±11 | 60 | | | 40 | | ^[8]^ |
| Chimeric MaSp1*^+/-^* | 95 | - | 371.5±27 | 32.2±4.6 | | | 84.8±14.4 | | ^[9]^ |
| Flag/MaSp2 | 62 | - | 92.0±21 | 43.8±26.9 | | | 31.4±22.0 | | ^[10]^ |
| chimeric (188)_6_ | 73 | - | 28.07±8.08 | 26.55±6.52 | | | 6.09±1.48 | | ^[11]^ |
| MaSp1/MaSp2 4:1 | 59 | - | 37.63±20.35 | 53.87±67.95 | | | 17.36±20.1 | |  |
| MaSp1 N16C | 66 | - | 290 | 47.1 | | | 100.9 | | ^[12]^ |
| GFY-PSD | 54 | - | 55.73 | 84.5 | | | 89 | | ^[13]^ |
| G-PSD | 66 | - | 150.58±31.32 | 132.8±76.3 | | | 61.55±48 | |  |
| 96-mer | 284.9 | - | 508±108 | 15±5 | | | 134.57 | | ^[14]^ |
| 192-mer | 556 | - | 1020 | 18±6 | | | 114±51 | | ^[15]^ |
| MaSp2/SpiCE-NMa1 | 50 | - | 200 | 15 | | | 25 | | ^[16]^ |
| NRRRC/TiO_2_/FA | 93 | - | 167 | 197 | | | 249 | | ^[17]^ |
| NR_7_-C | 35 | + | 40 | 3 | | | 3.9 | | ^[18]^ |
| NT2RepCT | 33 | + | 80 | 37 | | | 40 | | ^[19]^ |
| N16C | 65 | - | 65 | 9 | | | 4.3 | | ^[20]^ |
| NT2RepCT | 33 | + | 110 | 113 | | | 90 | | ^[21]^ |
| MaSpI64 | 169 | - | 169 | 59 | | | 75 | | ^[22]^ |
| NT-MaSp1s-CT | 42 | + | 53 | 97 | | | 3.6 | | ^[23]^ |
| NTW_4_CT | 104 | - | 245 | 28 | | | 51 | | ^[24]^ |
| N1L(AQ)_24_NR3 | 134 | - | 308±131 | 54±15 | | | 90±29 | | ^[25]^ |
| RSSP (FA) | 210 | - | 286±13 | 31±4 | | | n.s. | | ^[26]^ |
| NRRRC | 92 | - | 119.00±3.56 | 70±7 | | | 62.74±4.59 | | ^[27]^ |
| *A. trifasciata* dragline | 272±14 | + | 890±130 | ~30 | | | 100±40 | | ^[28]^ |
| *A. diadematus* dragline | 250-350 | + | 1183±334 | 24±8 | | | 167±65 | | ^[29]^ |
| *N. clavipes* dragline | 200-350 | + | 965±217 | ~30 | | | 111±30 | |  |

Table S3. Mechanical properties of Ori fibers and C1 fibers.

|  | **Strain at break (%)** | **Strength (MPa)** | **Young's Modulus (GPa)** | **Toughness (MJ/m^3^)** |
| --- | --- | --- | --- | --- |
| Ori_CS fibers | 33.72 ± 6.41 | 99.62 ± 6.39 | 5.41 ± 1.72 | 26.96 ± 3.72 |
| Ori_MS fibers | 45.85 ± 2.61 | 316.95 ± 42.10 | 7.15 ± 1.02 | 97.40 ± 16.76 |
| C1_CS fibers | 38.11 ± 6.26 | 223.62 ± 29.38 | 10.85 ± 3.81 | 66.44 ± 15.43 |
| C1_MS fibers | 52.78 ± 2.27 | 531.76 ± 33.46 | 12.31 ± 3.58 | 182.06 ± 6.67 |
| C1_CS  (DTT-treated) | 33.95 ± 1.49 | 105.10 ± 6.47 | 1.82 ± 0.46 | 28.54 ± 2.15 |
| C1_MS  (DTT-treated) | 34.71 ± 3.16 | 258.42 ± 31.98 | 5.06 ± 1.67 | 56.37 ± 8.88 |
| C1_MS  (air-oxidized) | 38.96 ± 5.02 | 401.82 ± 59.03 | 4.68 ± 1.01 | 87.88 ± 23.81 |

**Reference**

[1] H. Venkatesan, J. Chen, H. Liu, Y. Kim, S. Na, W. Liu, J. Hu, Artificial spider silk is smart like natural one: having humidity-sensitive shape memory with superior recovery stress. *Materials Chemistry Frontiers.* **2019**, *3*, 2472.

[2] M. A. Rapsomaniki, P. Kotsantis, I.-E. Symeonidou, N.-N. Giakoumakis, S. Taraviras, Z. Lygerou, easyFRAP: an interactive, easy-to-use tool for qualitative and quantitative analysis of FRAP data. *Bioinformatics.* **2012**, *28*, 1800.

[3] S. Keten, M. J. Buehler, Nanostructure and molecular mechanics of spider dragline silk protein assemblies. *Journal of The Royal Society Interface.* **2010**, *7*, 1709.

[4] N. Schmid, A. P. Eichenberger, A. Choutko, S. Riniker, M. Winger, A. E. Mark, W. F. van Gunsteren, Definition and testing of the GROMOS force-field versions 54A7 and 54B7. *European Biophysics Journal.* **2011**, *40*, 843.

[5] D. Frishman, P. Argos, Knowledge-based protein secondary structure assignment. *Proteins.* **1995**, *23*, 566.

[6] M. Saric, L. Eisoldt, V. Döring, T. Scheibel, Interplay of Different Major Ampullate Spidroins during Assembly and Implications for Fiber Mechanics. *Advanced Materials.* **2021**, *33*, 2006499.

[7] T. Arndt, G. Greco, B. Schmuck, J. Bunz, O. Shilkova, J. Francis, N. M. Pugno, K. Jaudzems, A. Barth, J. Johansson, A. Rising, Engineered Spider Silk Proteins for Biomimetic Spinning of Fibers with Toughness Equal to Dragline Silks. *Advanced Functional Materials.* **2022**, *32*.

[8] J. Li, Y. Zhu, H. Yu, B. Dai, Y. S. Jun, F. Zhang, Microbially Synthesized Polymeric Amyloid Fiber Promotes beta-Nanocrystal Formation and Displays Gigapascal Tensile Strength. *ACS Nano.* **2021**.

[9] J. Xu, Q. Dong, Y. Yu, B. Niu, D. Ji, M. Li, Y. Huang, X. Chen, A. Tan, Mass spider silk production through targeted gene replacement in <i>Bombyx mori</i>. *Proceedings of the National Academy of Sciences.* **2018**, *115*, 8757.

[10] F. Teulé, B. Addison, A. R. Cooper, J. Ayon, R. W. Henning, C. J. Benmore, G. P. Holland, J. L. Yarger, R. V. Lewis, Combining flagelliform and dragline spider silk motifs to produce tunable synthetic biopolymer fibers. *Biopolymers.* **2012**, *97*, 418.

[11] B. An, J. E. Jenkins, S. Sampath, G. P. Holland, M. Hinman, J. L. Yarger, R. Lewis, Reproducing Natural Spider Silks’ Copolymer Behavior in Synthetic Silk Mimics. *Biomacromolecules.* **2012**, *13*, 3938.

[12] C. F. Hu, Z. G. Qian, Q. Peng, Y. Zhang, X. X. Xia, Unconventional Spidroin Assemblies in Aqueous Dope for Spinning into Tough Synthetic Fibers. *ACS Biomater Sci Eng.* **2021**, *7*, 3608.

[13] S. L. Adrianos, F. Teulé, M. B. Hinman, J. A. Jones, W. S. Weber, J. L. Yarger, R. V. Lewis, Nephila clavipes Flagelliform Silk-Like GGX Motifs Contribute to Extensibility and Spacer Motifs Contribute to Strength in Synthetic Spider Silk Fibers. *Biomacromolecules.* **2013**, *14*, 1751.

[14] X.-X. Xia, Z.-G. Qian, C. S. Ki, Y. H. Park, D. L. Kaplan, S. Y. Lee, Native-sized recombinant spider silk protein produced in metabolically engineered Escherichia coli results in a strong fiber. *Proceedings of the National Academy of Sciences.* **2010**, *107*, 14059.

[15] C. H. Bowen, B. Dai, C. J. Sargent, W. Bai, P. Ladiwala, H. Feng, W. Huang, D. L. Kaplan, J. M. Galazka, F. Zhang, Recombinant Spidroins Fully Replicate Primary Mechanical Properties of Natural Spider Silk. *Biomacromolecules.* **2018**, *19*, 3853.

[16] N. Kono, H. Nakamura, M. Mori, Y. Yoshida, R. Ohtoshi, A. D. Malay, D. A. Pedrazzoli Moran, M. Tomita, K. Numata, K. Arakawa, Multicomponent nature underlies the extraordinary mechanical properties of spider dragline silk. *Proceedings of the National Academy of Sciences.* **2021**, *118*, e2107065118.

[17] H. Zhu, Y. Sun, T. Yi, S. Wang, J. Mi, Q. Meng, Tough synthetic spider-silk fibers obtained by titanium dioxide incorporation and formaldehyde cross-linking in a simple wet-spinning process. *Biochimie.* **2020**, *175*, 77.

[18] W. Finnigan, A. D. Roberts, C. Ligorio, N. S. Scrutton, R. Breitling, J. J. Blaker, E. Takano, The effect of terminal globular domains on the response of recombinant mini-spidroins to fiber spinning triggers. *Scientific Reports.* **2020**, *10*, 10671.

[19] G. Greco, J. Francis, T. Arndt, B. Schmuck, G. B. F, A. Barth, J. Johansson, M. P. N, A. Rising, Properties of Biomimetic Artificial Spider Silk Fibers Tuned by PostSpin Bath Incubation. *Molecules.* **2020**, *25*.

[20] J. Cheng, C.-F. Hu, C.-Y. Gan, X.-X. Xia, Z.-G. Qian, Functionalization and Reinforcement of Recombinant Spider Dragline Silk Fibers by Confined Nanoparticle Formation. *ACS Biomaterials Science & Engineering.* **2022**, *8*, 3299.

[21] B. Schmuck, G. Greco, F. G. Bäcklund, N. M. Pugno, J. Johansson, A. Rising, Impact of physio-chemical spinning conditions on the mechanical properties of biomimetic spider silk fibers. *Communications Materials.* **2022**, *3*, 83.

[22] Q. Jin, F. Pan, C.-F. Hu, S. Y. Lee, X.-X. Xia, Z.-G. Qian, Secretory production of spider silk proteins in metabolically engineered Corynebacterium glutamicum for spinning into tough fibers. *Metabolic Engineering.* **2022**, *70*, 102.

[23] C. Zhang, J. Mi, H. Qi, J. Huang, S. Liu, L. Zhang, D. Fan, Engineered a novel pH-sensitive short major ampullate spidroin. *International Journal of Biological Macromolecules.* **2020**, *154*, 698.

[24] Y. Zhou, A. Rising, J. Johansson, Q. Meng, Production and Properties of Triple Chimeric Spidroins. *Biomacromolecules.* **2018**, *19*, 2825.

[25] A. Heidebrecht, L. Eisoldt, J. Diehl, A. Schmidt, M. Geffers, G. Lang, T. Scheibel, Biomimetic Fibers Made of Recombinant Spidroins with the Same Toughness as Natural Spider Silk. *Advanced Materials.* **2015**, *27*, 2189.

[26] T. Asakura, H. Matsuda, A. Naito, Y. Abe, Formylation of Recombinant Spider Silk in Formic Acid and Wet Spinning Studied Using Nuclear Magnetic Resonance and Infrared Spectroscopies. *ACS Biomaterials Science & Engineering.* **2022**, *8*, 2390.

[27] H. Zhu, A. Rising, J. Johansson, X. Zhang, Y. Lin, L. Zhang, T. Yi, J. Mi, Q. Meng, Tensile properties of synthetic pyriform spider silk fibers depend on the number of repetitive units as well as the presence of N- and C-terminal domains. *International Journal of Biological Macromolecules.* **2020**, *154*, 765.

[28] G. R. Plaza, J. Pérez-Rigueiro, C. Riekel, G. B. Perea, F. Agulló-Rueda, M. Burghammer, G. V. Guinea, M. Elices, Relationship between microstructure and mechanical properties in spider silk fibers: identification of two regimes in the microstructural changes. *Soft Matter.* **2012**, *8*, 6015.

[29] G. Greco, H. Mirbaha, B. Schmuck, A. Rising, N. M. Pugno, Artificial and natural silk materials have high mechanical property variability regardless of sample size. *Scientific Reports.* **2022**, *12*, 3507.
